# Producing Magnetic Nanocomposites from Paper Sludge for the Adsorptive Removal of Pharmaceuticals from Water—A Fractional Factorial Design

**DOI:** 10.3390/nano11020287

**Published:** 2021-01-22

**Authors:** Luciana S. Rocha, Érika M. L. Sousa, María V. Gil, João A. B. P. Oliveira, Marta Otero, Valdemar I. Esteves, Vânia Calisto

**Affiliations:** 1 Department of Chemistry and CESAM, University of Aveiro, 3810-193 Aveiro, Portugal; erikamsousa@ua.pt (É.M.L.S.); jabpo@ua.pt (J.A.B.P.O.); valdemar@ua.pt (V.I.E.); vania.calisto@ua.pt (V.C.); 2 Instituto de Ciencia y Tecnología del Carbono, INCAR-CSIC, Francisco Pintado Fe 26, 33011 Oviedo, Spain; victoria.gil@incar.csic.es; 3 Department of Environment and Planning and CESAM, University of Aveiro, 3810-193 Aveiro, Portugal; marta.otero@ua.pt

**Keywords:** activated carbon, adsorption, aquatic environment, emerging contaminants, micro-organic contaminants, magnetic materials, multivariate analysis

## Abstract

In view of a simple after-use separation, the potentiality of producing magnetic activated carbon (MAC) by intercalation of ferromagnetic metal oxide nanoparticles in the framework of a powder activated carbon (PAC) produced from primary paper sludge was explored in this work. The synthesis conditions to produce cost effective and efficient MACs for the adsorptive removal of pharmaceuticals (amoxicillin, carbamazepine, and diclofenac) from aqueous media were evaluated. For this purpose, a fractional factorial design (FFD) was applied to assess the effect of the most significant variables (Fe^3+^ to Fe^2+^ salts ratio, PAC to iron salts ratio, temperature, and pH), on the following responses concerning the resulting MACs: Specific surface area (*S*_BET_), saturation magnetization (*M*_s_), and adsorption percentage of amoxicillin, carbamazepine, and diclofenac. The statistical analysis revealed that the PAC to iron salts mass ratio was the main factor affecting the considered responses. A quadratic linear regression model A = f(*S*_BET_, *M*_s_) was adjusted to the FFD data, allowing to differentiate four of the eighteen MACs produced. These MACs were distinguished by being easily recovered from aqueous phase using a permanent magnet (*M*_s_ of 22–27 emu g^−1^), and their high *S*_BET_ (741–795 m^2^ g^−1^) were responsible for individual adsorption percentages ranging between 61% and 84% using small MAC doses (35 mg L^−1^).

## 1. Introduction

Over the past decade, pharmaceuticals have been recognized as potential toxic environmental contaminants and, as a result, their occurrence in aquatic environments gained considerable attention worldwide. Resulting from their massive consumption, substantial amounts of pharmaceuticals in both their unchanged and metabolized forms are directly or indirectly discarded into aquatic systems. Therefore, concentration levels of ng L^−1^ to μg L^−1^ can be commonly found in surface water and groundwater [1,2,3] and some studies have even reported the occurrence of pharmaceuticals at mg L^−1^ levels [4,5]. Given the detected values and due to their persistence in natural waters, pharmaceuticals pose a long-term menace to aquatic organisms and can induce undesirable effects on both humans and ecosystems [1,2,6,7]. In order to address emerging concern regarding the contamination by pharmaceuticals, Directive 2013/39 EU set the necessity of studying their environmental risks and of protecting the aquatic environment and human health, simultaneously highlighting that the development of innovative cost effective water treatment technologies should be stimulated [8]. 

Several approaches have been investigated for the removal of pharmaceuticals from waters (e.g., biological treatments, reverse osmosis, nanofiltration, ozonation, and advanced oxidation processes) [9]. Among them, adsorption is a well-researched technique, and its recognition lies in the low initial investment needed, the operational simplicity and its versatility [1]. Carbonaceous adsorbents, in particular powdered activated carbon (PAC), are usually preferred for the adsorptive removal of pharmaceuticals from waters, due to their porous structure and their high specific surface area (*S*_BET_) [1,10,11]. The production of PAC using industrial and agricultural wastes as precursors has been focus of special attention, in a way to increase the sustainability of these carbon materials [10]. Pulp and paper mill sludge is an example of a widely produced waste (50 kg of dry sludge per tonne of paper), which economic and sustainable management is a continuous challenge for the industry. Given the cellulosic nature of this residue, it presents the adequate properties to be used as precursor of carbonaceous adsorbents. In this sense, the application of paper mill sludge-derived adsorbents for water remediation has been explored by several authors regarding the removal of pharmaceuticals [12,13,14,15], and other organic [16,17] and inorganic [18] contaminants from waters. Along with the use of alternative precursors, aiming to generalize the application and increase the potential of carbonaceous adsorbents, a lot of research work has been devoted to their structural and chemical modification. In the last decade, the anchorage of ferromagnetic metal oxides (e.g., iron oxides) on its carbon matrix has arisen as a suitable solution to overcome difficulties in the after-use separation of PAC [19,20]. The inclusion of magnetic nanoparticles on PAC allows for the recovery of the adsorbent using an external magnetic field, so avoiding the filtration and/or centrifugation operations usually needed for PAC separation. In this way, the use of magnetic activated carbon (MAC) reduces the overall process cost and complexity [20,21]. 

Different synthesis routes can be used to produce MAC (e.g., co-precipitation, oxidative hydrolysis of ferrous salts, thermochemical and mechanical treatments), but co-precipitation is typically the selected method, owing to its low cost and simplicity [20,22]. The magnetically active components usually introduced in PAC framework by co-precipitation are magnetite (Fe_3_O_4_) and maghemite (ɣ-Fe_2_O_3_). Anyhow, the magnetic, textural and morphologic properties imparted to MAC are highly dependent on the concentration of ferric (Fe^3+^) and/or ferrous (Fe^2+^) salts, the alkaline conditions (concentration of OH^−^) [23], the synthesis temperature [23,24] used to produce iron nanoparticles and also the ratio between PAC and iron salts [25]. Therefore, the management of the above-mentioned experimental parameters has been shown to be crucial to achieve the desired features in terms of size and distribution of nanoparticles [24,26], magnetic properties (saturation magnetization, *M*_s_) and *S*_BET_ of the materials [27]. However, to the best of our knowledge, the combined effect of these synthesis conditions and their impact on the features and adsorptive performance of waste-based MACs have not been assessed yet. 

The aim of this work was to determine the conditions to produce efficient magnet-responsive nanocomposite materials by in-situ iron oxide co-precipitation onto PAC prepared from paper mill sludge for application in the adsorption of pharmaceuticals from water. For that purpose, a fractional factorial design (FFD) was employed to evaluate the effect of production variables (Fe^3+^:Fe^2+^ molar ratio, PAC:Fe salts mass ratio, reaction temperature, and pH conditions) on the properties (*S*_BET_ and *M*_s_) and adsorptive performance (pharmaceutical percentage of adsorption, *A*) of the resulting waste-based MACs. For the latter, pharmaceuticals from three different therapeutic classes, namely amoxicillin (AMX, antibiotic), carbamazepine (CBZ, antiepileptic), and sodium diclofenac (DCF, non-steroidal anti-inflammatory drug) were selected as adsorbate models. After selecting the most appropriate production conditions, the morphology, composition and surface structure of the selected MACs were assessed by scanning electron microscopy (SEM), X-ray fluorescence (XRF), and X-ray photoelectron spectroscopy (XPS) analyses, respectively.

## 2. Materials and Methods

### 2.1. Reagents and Chemicals

The reagents used in the present work were of analytical grade. Potassium hydroxide (KOH, LABCHEM, ≥86%) was used in the chemical activation of primary paper mill sludge and in the synthesis of MAC by the co-precipitation method. Hydrochloric acid (HCl, AnalaR NORMAPUR, 37%) was used in the washing process of PAC. Ferric chloride hexahydrate (FeCl_3_·6H_2_O, >99%) and ferrous sulphate heptahydrate (FeSO_4_·7H_2_O, >99%) were purchased from Chem-Lab. The buffer solutions used for the pH meter calibration, with pH values of 4.01 ± 0.01, 7.01 ± 0.01, and 10.1 ± 0.01, were acquired from Hanna Instruments. The pH adjustments were performed using a solution of ca. 0.5 mol L^−1^ KOH. Adsorption studies were performed with three pharmaceuticals: Carbamazepine (C_15_H_12_N_2_O 99%, Sigma-Aldrich, St. Louis, MS, USA–EUA), sodium diclofenac salt (C_14_H_10_Cl_2_NNaO_2_, TCI, >98%) and amoxicillin tri-hydrate (C_16_H_25_N_3_O_8_S > 98%, TCI, Tokyo–Japan). The chemical structures and physico-chemical properties of these pharmaceuticals are depicted in Table S1 of Supplementary Materials. For micellar electrokinetic chromatography (MEKC) analyses, the following reagents were used: Hexadimethrine bromide ((C_13_H_30_Br_2_N_2_)_n_ 95%, Sigma) for capillary coating, ethylvanillin (C_2_H_5_OC_6_H_3_(OH)CHO 99%, Sigma-Aldrich,) as internal standard, and sodium tetraborate decahydrate (Na_2_B_4_O_7_·10H_2_O, Riedel-de-Haën, Seelze–Germany) and sodium dodecyl sulfate (CH_3_(CH_2_)_11_OSO_3_Na, PanReac, PA-ACS, Barcelona, Spain) as separation buffer. All solutions were prepared in ultrapure water (18.2 MΩ cm^−1^, PURELAB flex 4 system, ELGA VEOLIA, High Wycombe, UK).

### 2.2. Preparation of Powdered Activated Carbon 

Primary sludge (PS) from pulp and paper mill industry was used as precursor to prepare PAC, according to the optimized experimental conditions determined by Jaria et al. (2019) [28]. PS was collected from a pulp and paper mill that employs the kraft elemental chlorine free production process and uses eucalyptus (*Eucalyptus globulus*) wood. Briefly, PS was first impregnated with KOH activating agent using a 1:1 *w*/*w* ratio (batches of 15 g of PS with 15 g of KOH in 50 mL of distilled water), under ultrasonic stirring for 1 h and left to dry at room temperature in a laboratory fume hood. The dried material was then pyrolyzed in porcelain crucibles at 800 °C in a muffle for 150 min (heating rate of 10 °C min^−1^), under N_2_ atmosphere. The carbonized material was washed with 1.0 M HCl (for ashes and KOH removal) and distilled water (until neutral pH of the leachate was reached) and dried overnight at 100 °C. Finally, the material was crushed in order to obtain a fine homogenous powder [28]. 

### 2.3. Preparation of Magnetic Activated Carbon 

The loading of iron oxide magnetic nanoparticles onto PAC to produce MACs was performed by co-precipitation. The magnetic nanoparticles were synthetized by alkaline co-precipitation of FeCl_3_·6H_2_O and FeSO_4_·7H_2_O salts, and to avoid the formation of non-magnetic forms of iron oxides, the reaction was conducted in inert atmosphere. The systematic synthesis of MACs is described according to the following procedure. Firstly, the ultrapure water used in the synthesis was degassed for 30 min with N_2_ to prevent from oxidation of iron salt solutions prior to MAC synthesis. A solution containing both FeCl_3_·6H_2_O (concentration interval between 0.022–0.073 mol L^−1^) and FeSO_4_·7H_2_O (concentration interval between 0.037−0.087 mol L^−1^) salts was prepared (for a total volume of 50 mL) and transferred to a glass reactor (during this step no color alteration was observed, nor the formation of a precipitate), followed by the addition of the PAC prepared in Section 2.2. The mixture was then heated to a pre-defined temperature, under oxygen-free conditions (N_2_ flow) and stirred at 100 rpm. These conditions were kept during the whole operation. A KOH solution (~0.5 mol L^−1^) was added dropwise (for a volume varying between 39 mL and 50 mL) to achieve the desired pH and the reaction was held for 1 h, keeping the defined temperature. After magnetic decantation, the supernatant (excess of alkali solution) was discarded, and the produced MACs were thoroughly washed with distilled water until neutral pH of the washing leachate was reached. The materials were then dried at 50 °C for 48 h, mechanically grinded and finally stored in a sealed container prior to their use. A total of 18 MAC materials were produced and the experimental conditions used, i.e., molar ratio between FeCl_3_·6H_2_O and FeSO_4_·7H_2_O salts (Fe^3+^:Fe^2+^), mass ratio between PAC and iron salts (PAC:Fe), reaction temperature and pH conditions, are summarized in Table 1.

### 2.4. Process Variables and Experimental Fractional Factorial Design 

An experimental fractional factorial design (FFD) (relationship between input factors (variables) and output effects (responses) in a process) was used to determine the optimum set of operational variables to produce MAC materials, since it allows to perform a systematic optimization of the process with a reduced number of experiments and depletion of resources.

#### 2.4.1. Factors

The process variables in the procedure above described (Section 2.3) for the production of MACs were: Molar ratio between FeCl_3_·6H_2_O and FeSO_4_·7H_2_O salts (Fe^3+^:Fe^2+^), mass ratio between PAC and iron salts (PAC:Fe), reaction temperature and pH conditions, for which the considered values are depicted in Table 1. The test hypothesis was to investigate if each one of the referred variables had a significant impact on the characteristics and adsorptive performance of the resulting MAC (see Section 2.4.2 for selected responses). The variables and corresponding tested levels were chosen according to previous work of the group and other literature studies where such conditions were applied in an individual approach and not in a systematized way, hindering the possibility of a statistical analysis. Specifically, studies by İlbay et al. (2015) [29], Wong et al. (2016) [30], Badi et al. (2018) [26], Rai and Singh (2018) [19], and Lompe et al. (2018) [27] applied synthesis temperatures ranging from room temperature to 80 °C; Arya and Philip (2016) [31], Danalıoğlu et al. (2017) [32] and Rai and Singh (2018) [19] performed the MAC synthesis at pH ranging from 8 to 12; Fe^3+^:Fe^2+^ (*w*/*w*) salts ratio from 3.2:1 to 0.5:1 were explored by Castro et al. (2009) [33], İlbay et al. (2015) [29], Danalıoğlu et al. (2017) [32], Badi et al. (2018) [26] and Pereira et al. (2020) [15]; and PAC:Fe salts (*w*/*w*) ratio between 1:2 and 1:6 were addressed by İlbay et al. (2015) [29] and Pereira et al. (2020) [15].

The FFD design matrix was obtained by codifying the studied factors, i.e., Fe^3+^:Fe^2+^ molar ratio (χ_1_), PAC:Fe salts mass ratio (χ_2_), reaction temperature (χ_3_) and pH (χ_4_), as follows. The optimization of the MAC synthesis was performed using a fractional design with mixed levels: Three factors (χ_1_, χ_2_ and χ_3_) at three levels (3^3−1^) and a two-level factor (χ_4_). A total of eighteen MACs were produced according to the planned FFD ($N=3^{3-1\;}\times 2$). Each factor, when three levels are to be considered, is assigned to “low,” “medium” and “high”, being denoted as −1, 0, and +1, respectively, or “low” and “high” when only two levels are applied, as described in Table 1. 

#### 2.4.2. Responses

The magnitude and the direction of the factor effects on the response modelling were evaluated, using the following responses: *S*_BET_, *M*_s_ and percentage of adsorption of AMX, CBZ and DCF from aqueous solutions (*A*).

(i) Specific surface area

The *S*_BET_ of MACs was determined by N_2_ adsorption isotherms at −196 °C using a Micromeritics Instrument, Gemini VII 2380, after outgassing the materials overnight at 120 °C. The *S*_BET_ was calculated by means of Brunauer–Emmett–Teller equation [34] in the relative pressure range between 0.01 and 0.1. The N_2_ adsorption isotherms obtained for PAC and selected MACs from the FFD (MAC 4, MAC 7, MAC 11, and MAC 17) are presented in Figure S1, as Supplementary Materials. Additionally, the total micropore volume (*V*_mic_), the total pore volume (*V*_p_) and average pore width (*D*) were also determined. *V*_mic_ was determined by the Dubinin–Radushkevich equation [35], as for *V*_p_, this parameter was estimated from the amount of N_2_ adsorbed at a relative pressure of 0.99 and *D* was determined by using the following equation [12]:

*D* = 2 × *V*_p_/*S*_BET_ (1)

The pore size distribution was determined by Non-Local Density Functional Theory (NLDFT) analysis assuming slit pores.

(ii) Saturation magnetization

The magnetization measurements were conducted in a vibrating sample magnetometer (VSM EV9), with an applied magnetic field to a maximum of 22 kOe. By plotting the magnetic moment as a function of the applied magnetic field, it is possible to determine the *M*_s_ of each MAC, dividing the plateau found for the magnetic moment by the MAC mass (10 mg). Prior to the analysis, the instrument was calibrated with a disk of pure nickel and applying a magnetic field of c.a.1 Oe and with dispersion on the magnetic moment inferior to 0.5%.

(iii) Adsorption percentage

Individual solutions of AMX, CBZ and DCF with an initial concentration (*C*_i_) of 5 mg L^−1^ were prepared by dissolving a specific amount of each pharmaceutical in ultrapure water. Batch adsorption experiments were conducted in 50 mL polypropylene falcon tubes, into which were put into contact 40 mL of pharmaceutical solution (AMX, CBZ, or DCF, with no pH adjustment) with 1.4 mg of the corresponding MAC (dose of 35 mg L^−1^). Then, falcon tubes were shaken for 4 h (preliminary experiments with different MACs showed that such a contact time guarantees a situation of equilibrium) in an overhead shaker (Heidolph, Schwabach–Germany, Reax 2) at 80 rpm under controlled room temperature (25.0 ± 0.1 °C). After shaking during the defined time, the solutions were collected and filtered through 0.22 μm PVDF filters (Whatman) for the analytic determination of the remaining pharmaceutical concentration (*C*_f_). The analytic quantification of AMX, CBZ, and DCF was performed by micellar electrokinetic chromatography (MEKC), according to the procedure described in Section 2.5. Blank controls containing each pharmaceutical (*C*_i_ of 5 mg L^−1^ and without any MAC) were shaken during the same time as the adsorption experiments and used as reference for the calculation of adsorption percentages (*A*) for each pharmaceutical, using Equation (2):

(2) $$A=\frac{C_{0}-C_{f}}{C_{0}}\times 100$$

where *C*_f_ (mg L^−1^) is the remaining pharmaceutical concentration in the liquid phase of adsorption experiments at the end of shaking and *C*_0_ (mg L^−1^) is the concentration of pharmaceutical in the corresponding control experiments.

### 2.5. Analytic Quantification of Pharmaceuticals

The quantification of the pharmaceuticals in the aqueous solutions was carried out by MEKC, using a Beckman P/ACE MDQ (Fullerton, CA, USA) instrument, equipped with a UV-visible detection system. A silica capillary was dynamically coated according to the procedure described by Calisto et al. (2011) [36]. The electrophoretic separation was performed by direct polarity mode at 25 kV and 25 °C, during 2.5 min for AMX and DCF and during 3.0 min for CBZ. All samples and standard solutions were spiked with the internal standard ethylvanillin (final concentration of 3.34 mg L^−1^). The detection was monitored at 200 nm for DCF and AMX and 214 nm for CBZ. The separation buffer used was composed by 15 mmol L^−1^ of sodium tetraborate and 30 mmol L^−1^ of SDS for CBZ and AMX, and 15 mmol L^−1^ of sodium tetraborate and 50 mmol L^−1^ of SDS for DCF; the separation buffer was renewed every six runs. After each run, the capillary was washed with ultrapure water (60 s) and then with the separation buffer (90 s), at 20 psi. The determination of the calibration curve was carried out for each pharmaceutical using seven standard solutions with concentrations ranging between 0.500 mg L^−1^ and 5.00 mg L^−1^. All the analyses were performed in triplicate. The detection limit of the method (3σ), within the concentration range used, was 0.307 mg L^−1^, 0.263 mg L^−1^ and 0.289 mg L^−1^ for AMX, CBZ, and DCF, respectively.

### 2.6. Data Treatment

Analysis of variance (ANOVA) of the FFD results was applied to evaluate the significance of each experimental variable (χ_1_, χ_2_, χ_3_, and χ_4_) tested in the selected responses (*S*_BET_, *M*_s_, *A*_AMX_, *A*_CBZ_, and *A*_DCF_). The *p*-values were used as a tool to check the significance of each factor on the obtained responses, with a confidence level of 95% (*p*-values ≤ 0.05 indicated significant factors, while *p*-values > 0.05 indicated non-significant factors).

Furthermore, principal component analysis (PCA) and hierarchical cluster analysis (HCA) using centroid linkage method and Euclidean metric, were applied to the same data to identify patterns among the different MACs. Additionally, and using the loadings of the PC1 corresponding to the columns of the adsorption of AMX, CBZ, and DCF, an overall adsorption removal was defined, *A*_pooled_ (%), which was calculated by means of Equation (3):

*A*_pooled_ = 0.5294*A*_AMX_ + 0.5923*A*_CBZ_ + 0.6073*A*_DCF_. (3)

Also, a linear quadratic model of *A*_pooled_ versus *S*_BET_ and *M*_s_ was fitted and used to assess the optimal MAC regarding adsorption performance.

Matlab software R2019a (The MathWorks, Co., Natick, MA, USA) was used for all calculations and graphs.

### 2.7. Morphological and Chemical Characterization of Selected Materials

Materials selected from the previous FFD (based on the data analysis of Section 3.3) were further characterized, namely by the determination of the morphological and chemical composition. The morphological features of the selected MACs were evaluated by scanning electron microscopy (SEM) using a S4100 Hitachi (Tokyo, Japan) equipment and an electron acceleration voltage of 20 kV. Prior to the analysis, the samples were coated with a thin film of amorphous carbon. The following magnifications were used: 500×, 3000×, and 10,000×. The chemical composition on the MACs surface was determined by X-ray diffraction (XRD), X-ray fluorescence (XRF), and X-ray photoelectron spectroscopy (XPS). XRD measurements were performed at room temperature with a PANalytical Empyrean powder diffractometer using monochromated CuKα radiation (λ = 1.541° A) in the 80° 2θ range at 0.02° resolution, and 4000 acquisition points per step. The XRF analysis was performed using a Malvern Panalytical Axios spectrometer, under inert atmosphere (He) and applying a maximum voltage of 36.50 kV and 60 kV and a maximum current of 60 mA, 72 mA and 100 mA. The XPS spectra of the selected MACs were acquired in an Ultra High Vacuum (UHV) system with a base pressure of 2 × 10^–10^ mbar. The system is equipped with a hemispherical electron energy analyzer (SPECS Phoibos 150, Berlin, Germany), a delay-line detector and a monochromatic AlKα (1486.74 eV) X-ray source. High-resolution spectra were recorded at a normal emission take-off angle and with a pass-energy of 20 eV, which provides an overall instrumental peak broadening of 0.5 eV.

## 3. Results and Discussion

### 3.1. Fractional Factorial Design

**Figure 1:**
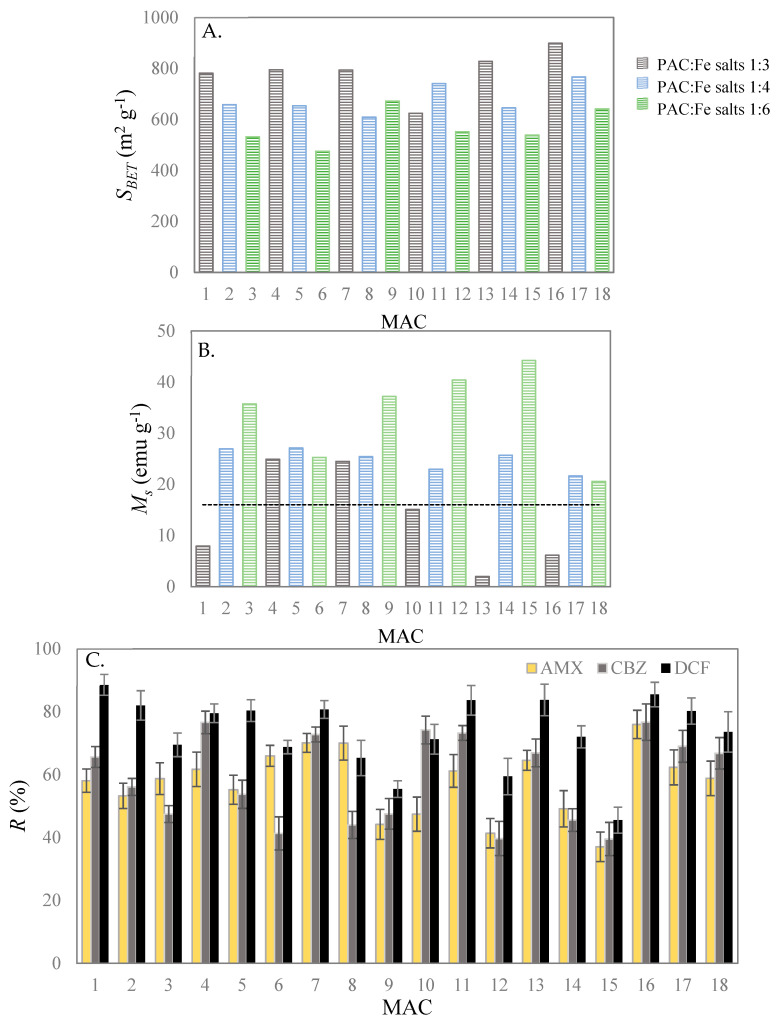
Values obtained for *S*_BET_ (**A**), *M*_s_ (**B**) and *A* of AMX, CBZ and DCF(**C**) for the 18 produced MACs. The line draw in (**B**) corresponds to the minimum *M*_s_ value (16 emu g^−1^) that assures an effective magnetic separation, according to [40].

The responses assessed in the FFD analysis, namely *S*_BET_, *M*_s_ and *A* for each of the considered pharmaceuticals, are shown in Figure 1 for the eighteen produced MACs.

Regarding the structural properties of the eighteen produced MACs, the *S*_BET_ ranged between 475 m^2^ g^−1^ and 899 m^2^ g^−1^, being these values lower than that obtained for the bare PAC (1438 m^2^ g^−1^). Still, *S*_BET_ of the here produced MACs are similar or even higher than most of values determined for waste-based magnetic carbons in the literature [22,37]. The lower *S*_BET_ of MACs when compared with PAC can be explained by the decrease and/or blockage of pores on PAC framework by the iron oxide nanoparticles produced by co-precipitation [27,38,39], and also by the very small *S*_BET_ values typically found in the literature for Fe_3_O_4_ (19 m^2^ g^−1^ [39]) and Fe_2_O_3_ (64 m^2^ g^−1^ [27]).

Furthermore, and according with the data presented in Table S2 of Supplementary Materials, the incorporation of magnetic iron nanoparticles onto the carbonaceous matrix was followed by a decrease on the total pore volume (*V*_p_) and micropore volume (*V*_mic_). The trend observed for the *V*_p_ and *V*_mic_ values was the same as that of *S*_BET_; the lowest *V*_p_ and *V*_mic_ were obtained for the MACs that had the lowest *S*_BET_, conversely the materials presenting the highest *S*_BET_ are characterized by having a porous structure. Also, it is possible to infer from these results the formation of well-developed pore structures in the eighteen produced MACs, with the micropore volume accounting for ca. 37% to 48% of the total pore volume. Regarding the pore size distribution, despite some differences that could be found (particularly for MACs with distinct *S*_BET_ values, as shown in Figure S2), typically all MACs exhibited identical profiles, with pores sizes ranging between 2 and 40 nm (mesopores), and with a higher incidence of pores between 2 nm and 15 nm. Additionally, macropores of ca. 63 nm were also detected in all MAC’s.

As for the magnetic properties of the produced MACs, the *M*_s_ values found for the overall MACs ranged between 2.0 emu g^−1^ and 44.2 emu g^−1^, the last one being closer to the values of 60 emu g^−1^ reported in the literature for Fe_3_O_4_ [25] and of 58 emu g^−1^ for Fe_2_O_3_ [27], both produced by the co-precipitation method. Besides that, as it may be seen in Figure 1, most of the produced MACs have *M*_s_ values over 16 emu g^−1^ (see the dashed line in Figure 1B) which according to Wang et al. (2014) [40], ensures a proper magnetic separation [40]. Furthermore, Figure 1A,B showed that, in some cases, decreasing the mass ratio of PAC to iron salts (factor χ_2_) and maintaining constant the Fe^3+^:Fe^2+^ molar ratio (factor χ_1_), *S*_BET_ decreases and consequently, *M*_s_ increases (e.g., MAC 1 to 3, MAC 13 to 15, and MAC 16 to 18).

This trend was also evidenced by other authors and was explained by the increase on the amount of magnetic iron oxide nanoparticles onto the AC framework [27,41,42]. However, this tendency was not shown by the remaining set of MAC materials (MAC 5 to 7, MAC 7 to 9 and MAC 10 to 12), probably because they are also affected by the temperature and pH of the reaction medium, also considered as variables in the synthesis of MACs.

MAC 16 (χ_1_ of 2:1 (*w*/*w*), χ_2_ of 1:3 molar ratio, χ_3_ of 60 °C and χ_4_ of 13.5), which was the material that presented the highest *S*_BET_ (899 m^2^ g^−1^), also displayed the highest adsorption efficiencies for all the pharmaceuticals (Figure 1C), with percentages of adsorption of (76.6 ± 4.5)% for AMX, (76.7 ± 5.8)% for CBZ and (85.5 ± 3.9)% for DCF. Despite these results, the low *M*_s_ of MAC 16 (6.2 emu g^−1^) resulted in a weak magnetic separation and recovery of the material from solution. On the other hand, MAC 15 presented a *M*_s_ of 44 emu g^−1^, which assures a fast and effective magnetic separation from aqueous media, but a relatively low *S*_BET_ (539 m^2^ g^−1^) that negatively affected the adsorptive performance of this material, with adsorption efficiencies of (45.5 ± 4.1)% for AMX, (39.5 ± 5.3)% for CBZ and (37.1 ± 4.7)% for DCF. Preliminary experiments showed that the bare magnetic iron oxide nanoparticles have almost no adsorption affinity towards these pharmaceuticals, and as for PAC, the adsorption of these three contaminants was almost complete (>95%).

### 3.2. Statistical Data Analysis

#### 3.2.1. Analysis of Variance

To evaluate the effects of the process factors on the studied responses, allowing for a more systematic analysis of the results, an ANOVA statistical analysis was performed, and the obtained results are depicted in Table S3 in Supplementary Materials.

The *p*-values above 0.05 obtained for Fe^3+^:Fe^2+^ molar ratio (χ_1_), reaction temperature (χ_3_) and reaction pH (χ_4_) suggest that none of these factors has a significant effect on the responses. For the factor χ_2_, the *p*-values below 0.05 (identified in bold in Table S3) attained for *S*_BET_ and *M*_s_, clearly indicate the effect of the mass ratio between PAC and iron salts used in the synthesis of MACs on both responses. Such an effect may be visualized in the relationship graphical representations displayed in Figure S3, as Supplementary Materials. The important role of the PAC:Fe ratio is in agreement with some previous studies, which refer the mass ratio between the carbonaceous precursor and the iron salts as one of the experimental conditions affecting the magnetic and structural properties of MACs produced by co-precipitation [27,41]. Moreover, the adsorption percentage of CBZ and DCF is also influenced by factor χ_2_ (*p*-values ≤ 0.05), and since the adsorptive removal of pharmaceuticals from aqueous solutions generally depends on and is positively correlated with *S*_BET_, this behavior was somehow expected [25,27,42]. This was not the case for AMX adsorption, since the *p*-values > 0.05 obtained from the ANOVA analysis point that the variables tested in the synthesis of MAC did not markedly affect this response.

**Figure 2:**
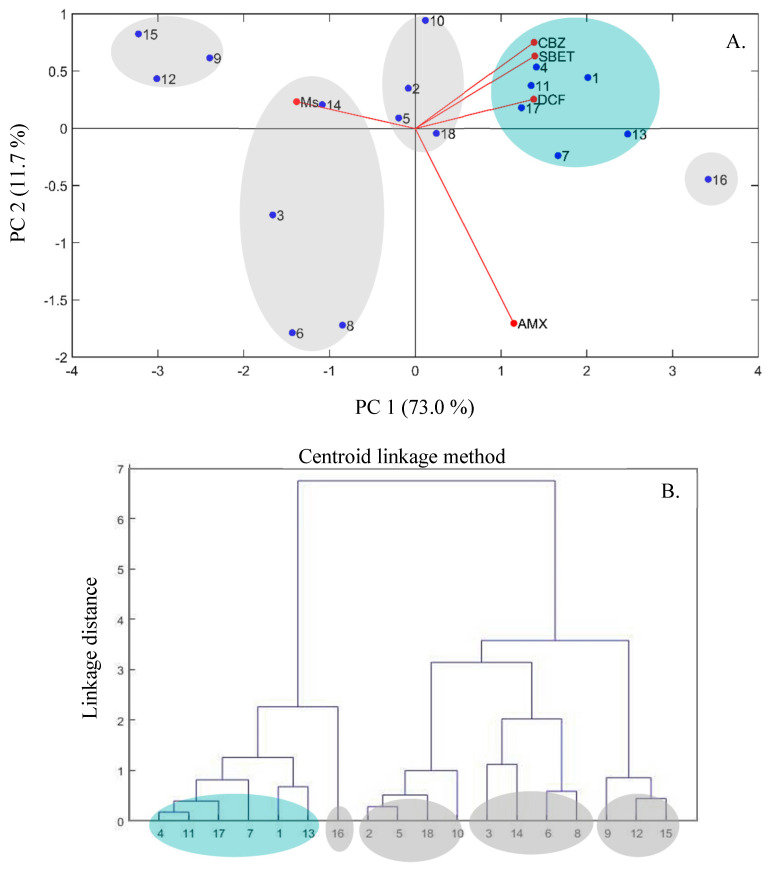
PCA score graph (**A**) and dendrogram of the hierarchical cluster analysis (HCA) results using centroid linkage method and Euclidean metric (**B**) of the eighteen MAC materials.

To identify patterns in the obtained responses, highlighting similarities and differences and allowing for the grouping of MACs, PCA and HCA analyses were performed, with the obtained results being shown in Figure 2. The score graph of the first two principal components (PC1 and PC2) explained a cumulative proportion of the data variance of 84.7%, corresponding to 73.0% and 11.7% respectively for PC1 and PC2 (Figure 2A). It is evident that *M*_s_ and *S*_BET_ values of the produced MAC follow opposite trends, which is related to the decrease in *S*_BET_ values resultant from the incorporation of the magnetic iron oxides nanoparticles in the carbonaceous framework (further information in Section 3.3). The adsorption percentages of CBZ and DCF and *S*_BET_ are grouped in the same quadrant, which reflects a strong and usually positive correlation between these responses. As for the adsorption of AMX, no correlation with the other responses was observed. However, other factors, namely the surface chemistry of MACs and the characteristics inherent to each pharmaceutical, have an important role in the removal efficiency. This might be a possible explanation for the fact that an increment in *S*_BET_ not always imply an increase in the adsorption percentage and the absence of correlation between *A*_AMX_ and the other responses.

From the PCA plot (Figure 2A) and according to the dendrogram of the HCA results using centroid linkage method and Euclidean metric (Figure 2B), five distinct clusters can be defined. Two groups presenting distinct and opposite *M*_s_ and *S*_BET_ values can be identified, the first one including MAC 15, MAC 12, and MAC 9 (group 1) and the second one, MAC 16 (group 2). Group 1 is characterized by MACs with the highest *M*_s_, with values varying between 37.2 emu g^−1^ and 44.2 emu g^−1^, the high amount of iron oxide magnetic nanoparticles dispersed on the PAC structure resulting in relatively low *S*_BET_, in particular for MAC 15 and MAC 12. Due to these low *S*_BET_ values, MAC 15, MAC 12, and MAC 9 exhibited the lowest adsorption percentages for AMX (between 37% and 44%), CBZ (between 40% and 48%) and DCF (between 46% and 59%). Group 2 is comprised solely by MAC 16, which has the highest *S*_BET_ value, and consequently the highest adsorption percentages towards AMX (65% to 76%), CBZ (67% to 77%) and DCF (between 84% and 86%). Three additional groups can be defined in the PCA biplot: Group 3 (MAC 1, MAC 4, MAC 7, MAC 11, MAC 13, and MAC 17), group 4 (MAC 2, MAC 5, MAC 10, and MAC 18) and group 5 (MAC 3, MAC 6, MAC 8, and MAC 14), most of them characterized by having intermediate values of *M*_s_ and *S*_BET_ and satisfactory *A* values towards the studied pharmaceuticals. Based on this overall analysis, MAC 4, MAC 11, and MAC 17 (sub-category of group 3) and MAC 2, MAC 5, and MAC 18 (sub-category of group 4) may be selected as appropriate materials for application in the adsorptive removal of pharmaceuticals, all of them having reasonably good responses (*S_BET_* > 640 m^2^ g^−1^, *M*_s_ > 20 emu g^−1^ and *A* > 50% for either AMX, CBZ or DFC, as shown in Figure 1).

#### 3.2.2. Quadratic Regression

In order to find the optimal MACs regarding adsorption performance, a quadratic linear regression model relating a weighted average of the adsorption of the three pharmaceuticals as a function of *M*_s_ and *S*_BET_ was fitted. The weights of each pharmaceutical are the components of the first eigenvector, i.e., the first principal component PC1 (73.0%). Equation (4) describes the relation between *S*_BET_, *M*_s_ and the overall adsorption removal (*A*_pooled_, calculated using Equation (3)), with a satisfactory correlation coefficient *R*^2^ of 0.891:

(4) $$A_{\mathrm{pooled}}=1.53\times 10^{-4}S_{\mathrm{BET}}{}^{2}-5.84\times 10^{-2}M_{s}{}^{2}-3.05\times 10^{-3}S_{\mathrm{BET}}M_{s}+4.26M_{s}-5.58\times 10^{-2}S_{\mathrm{BET}}+6.16\times 10^{1}\;$$

**Figure 3:**
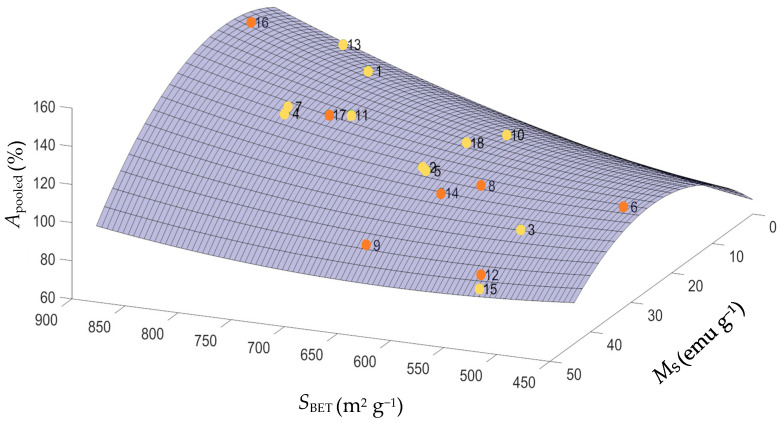
Three-dimensional plot of the three responses for the eighteen MAC materials.

From the 3D graphical representation of the three responses (*S*_BET_, *M*_s_ and *A*_pooled_), which is depicted in Figure 3, it is clear a positive correlation between *A*_pooled_ and *S*_BET_, and also that higher S_BET_ values typically result in MACs with lower *M*_s_, and vice-versa. This information corroborates the results from the PCA analysis, in which MAC 12 and MAC 15, and MAC 16 and MAC 13, present opposite trends regarding the *M*_s_ magnetic properties and both *S*_BET_ and *A*_pooled_.

Furthermore, based on this 3D graphical representation, it was possible to observe that MAC 4 and MAC 7 (χ_2_ of 1:3 and χ_4_ of 9.5) and MAC 11 and MAC 17 (χ_2_ of 1:4 and χ_4_ of 13.5), are the materials that gather the best compromise between magnetic properties and binding capacity towards the pharmaceuticals from different classes here considered. For these MAC, the *M*_s_ values ranged between 22 emu g^−1^ and 27 emu g^−1^, allowing their efficient separation from aqueous media, and the values of *S*_BET_ ranging between 741 m^2^ g^−1^ and 795 m^2^ g^−1^ were quite satisfactory, allowing the adsorption of 61% to 70%, 69% to 77%, and 80% to 84% for AMX, CBZ, and DCF, respectively.

### 3.3. Morphologic and Chemical Features of the Optimal MAC

**Figure 4:**
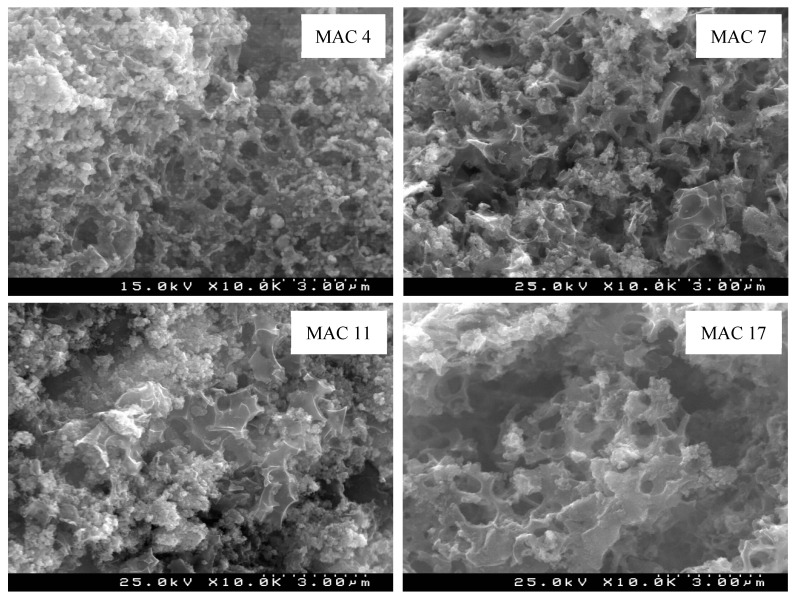
SEM images of the MAC 4, MAC 7, MAC 11, and MAC 17 using a magnification of 10,000×.

The morphologic features of MAC 4, MAC 7, MAC 11, and MAC 17 were evaluated by SEM, with the obtained images being represented in Figure 4. The micrographs of these four materials show small iron oxide nanoparticles of different shapes and sizes, distributed onto the surface of PAC. Besides that, it is clear from these images that the pores of the carbonaceous matrix of PAC are partially obstructed/blocked by the magnetic nanoparticles (see Figure S4, as SM) and this explains the decrease observed in both total pore volume (between 24% to 37%) and micropore volume (between 45% to 50%), and consequently in the *S*_BET_ values of these materials. Still, the SEM images of these MACs revealed the formation of well-developed porous structures, with the micropore volume accounting for ca. 39% to 46% of the total pore volume (according with the *S*_BET_ data provided in Table S2).

**Figure 5:**
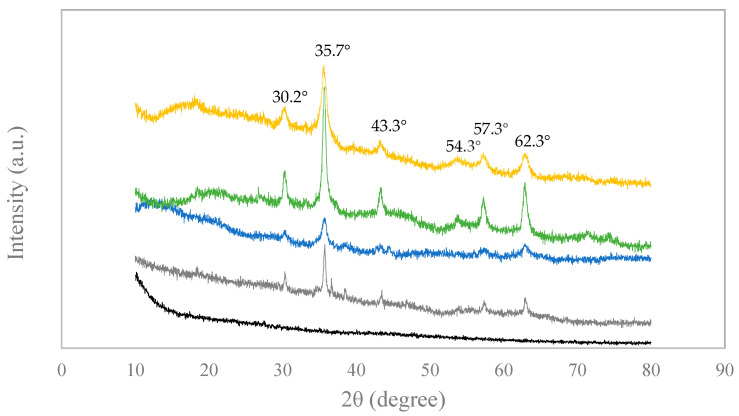
XRD patterns of PAC (-), MAC 4 (-), MAC 7 (-), MAC 11 (-), and MAC 17 (-).

The presence of magnetic iron oxides in the produced materials was evaluated by XRD, obtained results being depicted in Figure 5. The absence of peaks in the PAC highlighted the lack of a measurable crystallographic order, as this material is composed by non-graphitic and non-graphitizable carbon (Pereira et al., 2020) [15]. As for MAC 4, MAC 7, MAC 11, and MAC 17 all exhibited a characteristic XRD pattern with peaks at around 30.2°, 35.7°, 43.3°, 54.3°, 57.3°, and 62.9°, associated with the cubic spinel structure of magnetite and maghemite [15,32,43]. Although XRD results prove the existence of iron magnetic nanoparticles (magnetite and/or maghemite), they do not allow for distinguishing between these two crystalline components.

The elemental composition of PAC and the four MACs was evaluated by XRF (Table 2). The obtained data indicate that MACs are mainly composed by Fe (>95%), a value considerably higher than that of PAC (16%). These results support the successful incorporation of iron magnetic nanoparticles on the carbonaceous framework of PAC.

Evaluating their oxide composition, the results indicate the presence of Fe_2_O_3_ in MACs. Since these materials exhibited magnetic properties (*M*_s_ ranging between 22 emu g^−1^ and 27 emu g^−1^) it is likely that the iron oxide nanoparticles are in the form of maghemite (ɣ-Fe_2_O_3_), and not the non-magnetic form hematite (α-Fe_2_O_3_). Besides that, the occurrence of maghemite instead of magnetite might result from the incomplete removal of oxygen from the reaction medium (despite of the N_2_ flux used), contributing to the oxidation of magnetite (usually reported as being unstable in an oxidizing environment [43,44]). Other elements were also found in MACs, namely Si, Ca, S, K, Ti, Cl, and P and their presence could derive from the precursor itself and from the chemical activation (PAC production) and co-precipitation procedures, to which the precursor was subjected. Furthermore, the very low percentages found for Cl (<0.12%) and S (<0.75%), suggests the nearly total absence of unreacted iron sulphate/chloride salts in the carbon matrix (probably eliminated during the last stage of H_2_O washing) and that the Fe content was due to the presence of iron oxide nanoparticles in MAC’s composition. In the case of PAC, the Fe, Si, Ca, S, P, K, and Ti were detected with mass percentages varying between 4.0 and 22%, accounting for 96% of the elements detected. Just like in MACs, the presence of these elements in PAC’s composition can derive from both raw material and procedure applied to its production.

The surface chemical composition of MAC 4, MAC 7, MAC 11, and MAC 17 was further examined by XPS and compared with that of PAC. The XPS spectra obtained for PAC, MAC 4, MAC 7, MAC 11, and MAC 17 are presented in Figure S2, as Supplementary Materials. The XPS spectrum of PAC shows two major peaks, which are the C1s and O1s, while in the case of MAC 4, besides these two, Fe2p and Fe3s peaks were also identified. Other small peaks were also visible, namely Si2p, Si2s, and N1s for all the analyzed (Figure S5).

**Figure 6:**
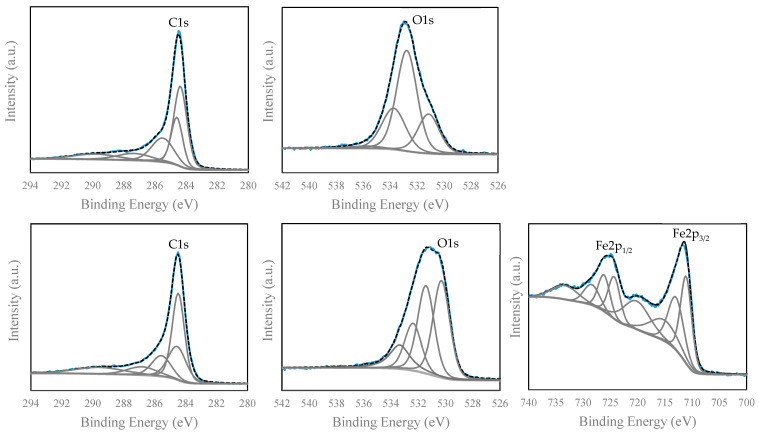
Deconvolution of C1s, O1s, and Fe2p XPS peaks of PAC and MAC 4: Experimental peak (-), adjusted peak (---) and the component groups (-).

The C1s, O1s and Fe2p peaks of MAC were deconvoluted and compared with those of PAC. Table 3 presents the component groups, binding energies and atomic concentrations of PAC and of the four MACs, while Figure 6 presents the deconvoluted C1s and O1s peaks of PAC and C1s, O1s, Fe2p peaks for MAC 4 (as for MAC 7, MAC 11 e MAC 17 the information is depicted in Figure S6, as Supplementary Materials).

As seen in Table 3, the deconvolution of the C1s region of PAC and the four selected MACs showed the presence of 5 peaks with binding energies ranging between 284 eV and 289 eV. These binding energies were assigned to the graphitic carbon sp^2^, the presence of C–C sp^3^ and C–H and other surface functional groups including C–O (ether and alcohol), C=O (carbonyl, quinones, and ketones) and O–C=O (carboxylic acids and carboxylic anhydride) [45,46]. In both PAC and MACs, the peaks with the lowest binding energies (graphitic C and C–C sp^3^/C–H) represent the prevailing peaks of C1s, except for MAC 7. By deconvoluting the O1s peak of PAC, a main peak at 533 eV appears and is attributed to O–H in hydroxyl groups and carbonyl (C=O) oxygen atoms in lactone and anhydrides. Besides that, two other peaks appear at 534 eV and 531 eV due to non-carbonyl oxygen atoms O–C=O in lactone and anhydrides and the carbonyl group and C=O in quinones; a small peak at 536 eV appears due to the contribution from the physiosorbed water [45,46]. As for MAC materials, the deconvolution of O1s results in an additional peak at 530 eV, resulting from Fe–O [42,47]. The other peaks at 531 eV, 533 eV, and 534 eV are attributed to the oxygen in the same functional groups as in PAC and no peaks were detected at ca. 536 eV. Regarding the surface chemistry, and from the functionalities identified in O1s and C1s deconvoluted peaks (Table 3), a decrease was observed in the percentage of carbonyl, carboxyl and hydroxyl groups in the four MACs when compared with PAC. Since these surface functional groups can act as binding sites, their content may have a significant impact on the adsorption of pharmaceuticals.

The XPS peak of Fe2p shows two peaks, one corresponding to Fe2p_3/2_ peak with a binding energy between 711.4 eV and 711.8 eV and the other to Fe2p_1/2_ appearing between 724.8 and 725.2 eV. The separation between these two peaks for the four MACs varies between 13.2 and 13.8 eV and the ratio between the area of Fe2p_3/2_ and Fe2p_1/2_ peaks is ca. 2. Besides that, a satellite peak of Fe2p_3/2_ appears at around 719.6 eV and is located just about 8 eV higher than the main Fe2p_3/2_ peak. According to Yamashita and Hayes (2008), the presence of this Fe2p_3/2_ satellite peak on the XPS spectra of MAC materials confirms the formation of Fe_2_O_3_ (maghemite) nanoparticles, since this peak does not appear in the XPS spectrum of Fe_3_O_4_ (magnetite) [48]. The formation of Fe_2_O_3_ instead of Fe_3_O_4_ corroborates the results obtained from XRF analysis. Besides, another peak appeared at 729.5 eV corresponding to the satellite peak for Fe2p_1/2_. The Fe2p of MAC 4 was deconvoluted, as showed in Figure 6. The Fe2p_3/2_ and Fe2p_1/2_ peaks and their corresponding satellites can be deconvoluted into Fe^2+^ and Fe^3+^ peaks and the lowest binding energies were always attributed to Fe^2+^ (see Table 3).

The total atomic percentage of each peak, along with the elemental composition, for PAC, MAC 4, MAC 7, MAC 11, and MAC 17 is listed in Table 3. The XPS data indicate contents of 74.9% of carbon and 17.8% of oxygen for the bare PAC. As for the composite MACs, the content of carbon decreased ca. 16% to 24% after loading the carbonaceous framework with iron oxide nanoparticles, and this variation was followed by an increase in both oxygen and iron contents, corresponding to the formation of iron oxides on their surface. Only trace amounts of nitrogen (<0.8%) were detected on the surface of all materials. Besides that, the XPS spectra identified the presence of a peak at around 102 eV, corresponding to the presence of SiO_2_ in all the five materials. The presence of silicon in these materials, with atomic concentrations varying between 4.7% and 6.3%, as stated before, might be due to the own composition of the carbonaceous precursor, namely PS, and the activation procedure applied to it.

### 3.4. Comparison with Waste-Based MAC in the Literature

The features of waste-based MAC 4, MAC 7, MAC 11 and MAC 17, regarding *M*_s_ and *S*_BET_, were compared with values reported in the literature (Table 4). The magnetic characteristics evidenced by the referred materials were typically superior than those obtained by other carbon materials containing magnetic iron oxide nanoparticles and applied in the adsorption of pharmaceuticals, with *M*_s_ values 1.4 to 4.8 times higher [30,41,42,47,49,50]. Besides that, their *S*_BET_ were also higher than the values reported in the literature for other MACs [30,41,42,47,49,50,51]. Differently, the MAC produced by Baghdadi et al. (2016) [25] presented a *S*_BET_ value 1.6 to 1.7 times higher than that of MAC 4, MAC 7, MAC 11, and MAC 17, yet, its *M*_s_ was ca. 5 times lower than for the MACs of the present work and, according to Wang et al. (2014) [40], insufficient to ensure an efficient magnetic separation (*M*_s_ < 16 emu g^−1^). In the case of the material obtained by Rai and Singh (2018) [19], despite of the 1.4–1.6 times higher *M*_s_, the *S*_BET_ was at least 2.6 lower than the values presented by the here selected MACs.

Very few information exists in the literature regarding the application of MAC in the removal of the AMX, CBZ, and DCF from aqueous media, which highlights the need for further research concerning this issue. Published data on the adsorption of these pharmaceuticals by different MACs are shown in Table S4 (depicted as Supplementary Materials) together with results obtained in this work. As it may be observed, all the *A* (%) in Table S4 are in the same order of magnitude, with the *A*_AMX_, *A*_CBZ_, and *A*_DCF_ of the MACs produced in this work being in the range of values published in the literature [18,36,39]. However, it must be pointed out that strict comparisons between the performance of the several materials cannot be done since different experimental conditions were used in each work. This is especially evident in the case of the pharmaceutical’s initial concentration and the adsorbent dose, with the lowest values being used in this study.

All the above considerations are indicative of the potential displayed by the waste-based MACs produced in this work, since they are capable of effectively adsorb AMX, CBZ and DCF from aqueous media, even when a small dose of material (35 mg L^−1^) is used, and are, simultaneously, easily and quickly recovered from the treated water by applying an external magnetic field. Due to the advantages associated with these materials, in particular their effective after-use separation, they may represent a suitable and sustainable alternative to PAC (avoiding the time consuming, costly, and inefficient separation stages) in water treatment. Considering such advantages, future work is to be carried out to further evaluate the performance of the selected MACs produced in this work, namely by determining the kinetic and equilibrium profiles towards the removal of different pharmaceuticals from WWTP effluents and exploring their regeneration/reutilization capacity by environmentally-friendly processes.

## 4. Conclusions

In the present work, nanocomposite materials exhibiting well-developed porous structures and containing nano-sized magnetic iron oxides in their framework were produced by a simple and cost-effective co-precipitation method and using primary paper mill sludge as PAC precursor. A FFD with mixed levels was applied and the combined effect of the synthesis conditions (Fe^2+^:Fe^3+^ salts molar ratio, PAC:Fe salts mass ratio, temperature, and pH) and their impact on both MAC’s features (*S*_BET_ and *M*_s_) and adsorptive removal of three pharmaceuticals (the antibiotic AMX, the antiepileptic CBZ and the non-steroidal anti-inflammatory DFC) was assessed. The data obtained from the multivariate analysis revealed that the mass ratio between PAC and the iron salts used in MAC’s production was the only factor significantly affecting all responses, except for the percentage of AMX adsorbed, which was not affected by any of the considered conditions. Furthermore, the obtained PCA score graph showed the definition of five distinct clusters and highlighted an opposite correlation between *S*_BET_ and *M*_s_ and a strong correlation between *A*_CBZ_, *A*_DCF_, and *S*_BET_ responses. As for the adsorption of AMX, no correlation with the other responses was observed. Finally, a 3D graphical analysis of *S*_BET_, *M*_s_ and *A*_pooled_ responses allowed for the selection of the production conditions used for MAC 4, MAC 7, MAC 11, and MAC 17 as the most favourable, with these materials exhibiting high *S*_BET_, large adsorption percentages (at relatively low MAC doses) ranging between 61–70%, 69–77%, and 80–84% for AMX, CBZ, and DCF, respectively, and still guaranteeing an efficient after-use recovery by magnetic separation (*M*_s_ between 22 emu g^−1^ and 27 emu g^−1^).

## Figures and Tables

**Table 1:** Experimental conditions (Fe^3+^:Fe^2+^ molar ratio, PAC:Fe mass ratio, reaction temperature and pH conditions), their codified variables and factor levels of the fractional factorial design (FFD) applied to optimize the production of the 18 magnetic activated carbons (MACs).

	Factors							
	Fe^3+^:Fe^2+^ (χ_1_)		PAC:Fe (χ_2_)		Temperature (χ_3_)		pH (χ_4_)	
MAC	Molar Ratio	Level	Mass Ratio	Level	°C	Level	pH	Level
1	1:4	1	1:3	−1	60	0	9.5	−1
2			1:4	0	60	0		
3			1:6	1	80	1		
4	1:3	0	1:3	−1	80	1	9.5	−1
5			1:4	0	60	0		
6			1:6	1	40	−1		
7	2:1	−1	1:3	−1	80	1	9.5	−1
8			1:4	0	40	−1		
9			1:6	1	40	−1		
10	1:4	1	1:3	−1	40	−1	13.5	1
11			1:4	0	40	−1		
12			1:6	1	80	1		
13	1:3	0	1:3	−1	40	−1	13.5	1
14			1:4	0	80	1		
15			1:6	1	60	0		
16	2:1	−1	1:3	−1	60	0	13.5	1
17			1:4	0	80	1		
18			1:6	1	60	0		

**Table 2:** Chemical composition (expressed in mass % of each element) of PAC, MAC 4, MAC 7, MAC 11, and MAC 17, obtained by XRF analysis.

	Fe	Si	Ca	S	K	Ti	Cl	P	Minor Elements ^1^
	(%)	(%)	(%)	(%)	(%)	(%)	(%)	(%)	(%)
PAC	16.2	19.1	17.8	21.5	10.8	7.0	4.0	1.4	2.2
MAC4	95.8	0.98	0.87	0.75	0.53	0.27	0.06	0.20	0.54
MAC7	95.0	0.91	0.86	0.58	1.24	0.30	0.12	0.19	0.80
MAC11	96.3	0.80	0.88	0.42	0.76	0.24	0.02	0.19	0.39
MAC17	95.2	0.61	1.00	0.32	1.54	0.24	0.06	0.18	0.85

^1^ Minor elements detected: Al, Br, Co, Cr, Cu, Mg, Mn, Mo, Ni, Sr, V, Zn, and Zr (PAC), Al, Cr, Cu, Mg, Mn, Ni, Pd, V, Zn, Zr (MAC 4, MAC 7, MAC 11, and MAC 17).

**Table 3:** Bond assignment (and respective abundance, in %), binding energies and total atomic percentages of C1s, O1s, Fe2p, Si2p, and N1s XPS peaks of PAC, MAC 4, MAC 7, MAC 11, and MAC 17.

		PAC		MAC 4		MAC 7		MAC 11		MAC 17	
Peak	Possible Bond Assignment	Bind. Energy	Abundance	Bind. Energy	Abundance	Bind. Energy	Abundance	Bind. Energy	Abundance	Bind. Energy	Abundance
	Binding	(eV)	(%)	(eV)	(%)	(eV)	(%)	(eV)	(%)	(eV)	(%)
	Graphitic C	284.4	40.5	284.4	40.4	284.2	26.4	284.3	36.0	284.3	38.8
	C–C sp^3^; C–H	284.6	21.2	284.5	22.9	284.5	36.6	284.6	23.1	284.6	21.0
C1s	C–O	285.5	20.3	285.6	15.0	285.2	18.2	285.5	18.5	285.2	26.2
	C=O	287.3	9.0	286.8	9.3	286.4	11.2	287.1	10.0	286.5	7.8
	O–C=O	289.8	9.0	289.6	12.3	289.0	7.5	289.9	12.4	288.9	6.2
	Total carbon percentage		74.9		62.6		57.2		60.8		58.8
	Fe–O	---	---	530.3	36.3	530.2	43.3	530.1	36.4	530.1	44.2
	C=O	531.1	21.4	531.4	32.5	531.2	24.2	531.0	24.8	530.9	22.3
O1s	O–H and C=O	532.8	52.2	532.4	17.6	532.1	14.5	531.8	20.1	531.7	13.9
	–C–O–C–	533.7	24.7	533.4	13.6	532.8	18.0	532.7	18.7	532.6	19.6
	Physiosorbed water	535.8	1.8	---	---	---	---	---	---	---	---
	Total oxygen percentage		17.8		24.4		28.1		25.5		26.5
	Fe2p_3/2_, Fe–O, Fe^2+^	---	---	711.1	20.7	710.8	21.2	710.9	21.0	710.7	19.9
	Fe2p_3/2_, Fe–O, Fe^3+^	---	---	713.0	17.8	712.6	16.3	712.6	14.8	712.4	16.2
	Fe2p_3/2_, Fe^2+^ satellite	---	---	715.2	12.8	714.9	12.1	714.7	12.4	714.5	12.1
Fe2p	Fe2_p3/2_, Fe^3+^ satellite	---	---	720.2	15.8	719.6	15.3	719.6	16.3	719.5	16.7
	Fe2p_1/2_, Fe–O, Fe^2+^	---	---	724.3	10.4	724.1	11.9	724.0	10.8	724.1	11.3
	Fe2p_1/2_, Fe–O, Fe^3+^	---	---	726.2	8.9	725.9	8.7	725.7	8.4	725.6	8.3
	Fe2p_1/2_, Fe^2+^ satellite	---	---	728.4	6.4	728.0	7.3	727.6	8.4	727.6	8.0
	Fe2p_1/2_, Fe^3+^ satellite	---	---	733.4	7.2	733.1	7.1	732.7	7.9	732.7	7.6
	Total iron percentage		0.0		6.6		8.7		8.4		9.5
Si2p	SiO_2_	103.8	6.3	102.2	5.0	102.1	5.3	101.8	4.8	101.7	4.7
N1s	Pyridinic N (N-6)	397.6	0.3	399.9	0.8	400.1	0.2	400.0	0.5	400.1	0.4

**Table 4:** Values of *M*_s_ and *S*_BET_ obtained for several MAC aimed at the adsorption of pharmaceuticals from water and produced by loading iron oxide nanoparticles onto different carbon precursors by different synthesis methods.

Carbon Precursor	MAC Synthesis	*S*_BET_ (m^2^ g^−1^)	*M*_s_ (emu g^−1^)	References
Paper mill sludge/KOH activation and pyrolysis	Co-precipitation	741–795	21.6–24.9	Present study
Commercial powder AC followed by HNO_3_ treatment	Co-precipitation	1241	5.1	[25]
Polyethylene terephthalate containers/pyrolysis and calcination with CO_2_	Co-precipitation	289	35.4	[19]
Commercial powder AC followed by treatment with basic steam	Co-precipitation	556	5.2	[30]
Sugarcane bagasse/NaOH activation	Co-precipitation	43	9.7	[47]
Coconut shell/H_2_SO_4_ activation and pyrolysis	Co-precipitation	335	15.8	[50]
Commercial PAC	Oxidative hydrolysis of Fe^II2+^ in alkaline media	666–556	2.3–9.8	[41]
Commercial AC	Oxidative hydrolysis of Fe^2+^ in alkaline media	535–652	2.0–14.8	[42]
Commercial PAC followed by HNO_3_ treatment	Thermochemical methods	671	6.9	[49]
Commercial granular AC	Ball milling	486	20.8	[51]

## Data Availability

Data is contained within the article and Supplementary Materials.

## Supplementary Materials

The following are available online at https://www.mdpi.com/2079-4991/11/2/287/s1, Table S1: Physical and chemical properties of amoxicillin tri-hydrate (AMX), carbamazepine (CBZ) and sodium diclofenac (DCF), Table S2: Values obtained for the specific surface area (*S*_BET_) and textural properties (total pore volume *V*_p_, micropore volume *V*_mic_, and average pore diameter *D*) of PAC and the eighteen MACs, Table S3: Results of ANOVA analysis: Sum of square (*SS*) and mean of square (*MS*) values, degree of freedom (*df*), F-test and the *p*-value (confidence level of 95%), Table S4: Values of A (%) obtained for AMX, CBZ, and DCF using MACs prepared using different carbon precursors, along with the experimental conditions used in the adsorption experiments (Ci of each pharmaceutical, dose of MAC, pH, temperature and contact time), Figure S1: N2 adsorption isotherms expressed as (A.) adsorption (cm^3^ STP g^−1^) vs relative pressure (p/p^0^) and (B.) adsorption (cm^3^ STP g^−1^) vs -log (p/p^0^) of the following materials: PAC, MAC 4, MAC 7, MAC 11, and MAC 17, Figure S2: Pore size distribution of PAC, MAC 3, MAC6, MAC 13, and MAC 16, Figure S3. Values obtained for SBET (A.) and Ms (B.) as a function of PAC:Fe salts ratio (*w*/*w*), Figure S4: SEM images of the MAC 7 using a magnification of 20,000 (A.) and 40,000× (B.), Figure S5: Overall XPS spectra of PAC, MAC 4, MAC 7, MAC 11, and MAC 17, Figure S6: Deconvolution of C1s, O1s, and Fe2p XPS peaks of MAC 7, MAC 11, and MAC 17: Experimental peak, adjusted peak and the component groups.

## References

[1] Akhtar J., Amin N.A.S., Shahzad K. (2015). A review on removal of pharmaceuticals from water by adsorption. Desalin. Water Treat..

[2] Ebele A.J., Abou-Elwafa Abdallah M., Harrad S. (2017). Pharmaceuticals and personal care products (PPCPs) in the freshwater aquatic environment. Emerg. Contam..

[3] Patel M., Kumar R., Kishor K., Mlsna T., Pittman C.U., Mohan D. (2019). Pharmaceuticals of emerging concern in aquatic systems: Chemistry, occurrence, effects, and removal methods. Chem. Rev..

[4] Fick J., Söderström H., Lindberg R.H., Phan C., Tysklind M., Larsson D.G.J. (2009). Pharmaceuticals and Personal Care Products in the Environment CONTAMINATION OF SURFACE, GROUND, AND DRINKING WATER FROM PHARMACEUTICAL PRODUCTION. Environ. Toxicol. Chem..

[5] Larsson D.G.J., de Pedro C., Paxeus N. (2007). Effluent from drug manufactures contains extremely high levels of pharmaceuticals. J. Hazard. Mater..

[6] Zenker A., Cicero M.R., Prestinaci F., Bottoni P., Carere M. (2014). Bioaccumulation and biomagnification potential of pharmaceuticals with a focus to the aquatic environment. J. Environ. Manag..

[7] Sui Q., Cao X., Lu S., Zhao W., Qiu Z., Yu G. (2015). Occurrence, sources and fate of pharmaceuticals and personal care products in the groundwater: A review. Emerg. Contam..

[8] European Parliament (2013). Directive 2013/39/EU of the European Parliament and of the Council of 12 August 2013 Amending Directives 2000/60/EC and 2008/105/EC as Regards Priority Substances in the Field of Water Policy.

[9] Verlicchi P., Galletti A., Petrovic M., Barceló D. (2010). Hospital effluents as a source of emerging pollutants: An overview of micropollutants and sustainable treatment options. J. Hydrol..

[10] Silva C.P., Jaria G., Otero M., Esteves V.I., Calisto V. (2018). Waste-based alternative adsorbents for the remediation of pharmaceutical contaminated waters: Has a step forward already been taken?. Bioresour. Technol..

[11] Krahnstöver T., Wintgens T. (2018). Separating powdered activated carbon (PAC) from wastewater-Technical process options and assessment of removal efficiency. J. Environ. Chem. Eng..

[12] Calisto V., Ferreira C.I.A., Santos S.M., Victoria M., Otero M., Esteves V.I. (2014). Production of adsorbents by pyrolysis of paper mill sludge and application on the removal of citalopram from water. Bioresour. Technol..

[13] Jaria G., Calisto V., Victoria M., Otero M., Esteves V.I. (2015). Journal of Colloid and Interface Science Removal of fluoxetine from water by adsorbent materials produced from paper mill sludge. J. Colloid Interface Sci..

[14] Oliveira G., Calisto V., Santos S.M., Otero M., Esteves V.I. (2018). Paper pulp-based adsorbents for the removal of pharmaceuticals from wastewater: A novel approach towards diversi fi cation. Sci. Total Environ..

[15] Pereira D., Rocha L.S., Gil M.V., Otero M., Silva N.J.O., Esteves V.I., Calisto V. (2020). In situ functionalization of a cellulosic-based activated carbon with magnetic iron oxides for the removal of carbamazepine from wastewater. Environ. Sci. Pollut. Res..

[16] Masomi M., Ghoreyshi A.A., Najafpour G.D., Mohamed A.R. (2014). Adsorption of Phenolic Compounds onto the Activated Carbon Synthesized from Pulp and Paper Mill Sludge: Equilibrium Isotherm, Kinetics, Thermodynamics and Mechanism Studies. Int. J. Eng. Trans. A Basics.

[17] Calace N., Nardi E., Petronio B.M., Pietroletti M. (2002). Adsorption of phenols by papermill sludges. Environ. Pollut..

[18] Battaglia A., Calace N., Nardi E., Petronio B.M., Pietroletti M. (2007). Reduction of Pb and Zn bioavailable forms in metal polluted soils due to paper mill sludge addition. Effects on Pb and Zn transferability to barley. Bioresour. Technol..

[19] Rai P., Singh K.P. (2018). Valorization of Poly (ethylene) terephthalate (PET) wastes into magnetic carbon for adsorption of antibiotic from water: Characterization and application. J. Environ. Manage..

[20] Siddiqui M.T.H., Nizamuddin S., Baloch H.A., Mubarak N.M., Al-Ali M., Mazari S.A., Bhutto A.W., Abro R., Srinivasan M., Griffin G. (2019). Fabrication of advance magnetic carbon nano-materials and their potential applications: A review. J. Environ. Chem. Eng..

[21] Wong S., Ngadi N., Inuwa I.M., Hassan O. (2018). Recent advances in applications of activated carbon from biowaste for wastewater treatment: A short review. J. Clean. Prod..

[22] Rocha L.S., Pereira D., Sousa É., Otero M., Esteves V.I., Calisto V. (2020). Recent advances on the development and application of magnetic activated carbon and char for the removal of pharmaceutical compounds from waters: A review. Sci. Total Environ..

[23] Gnanaprakash G., Mahadevan S., Jayakumar T., Kalyanasundaram P., Philip J., Raj B. (2007). Effect of initial pH and temperature of iron salt solutions on formation of magnetite nanoparticles. Mater. Chem. Phys..

[24] Petrova T.M., Fachikov L., Hristov J. (2011). The magnetite as adsorbent for some hazardous species from aqueous solutions: A review. arXiv.

[25] Baghdadi M., Ghaffari E., Aminzadeh B. (2016). Removal of carbamazepine from municipal wastewater effluent using optimally synthesized magnetic activated carbon: Adsorption and sedimentation kinetic studies. J. Environ. Chem. Eng..

[26] Badi M.Y., Azari A., Pasalari H., Esrafili A., Farzadkia M. (2018). Modification of activated carbon with magnetic Fe3O4 nanoparticle composite for removal of ceftriaxone from aquatic solutions. J. Mol. Liq..

[27] Lompe K.M., Vo Duy S., Peldszus S., Sauvé S., Barbeau B. (2018). Removal of micropollutants by fresh and colonized magnetic powdered activated carbon. J. Hazard. Mater..

[28] Jaria G., Patrícia C., Oliveira J.A.B.P., Santos S.M., Victoria M., Otero M., Calisto V., Esteves V.I. (2019). Production of highly efficient activated carbons from industrial wastes for the removal of pharmaceuticals from water—A full factorial design. J. Hazard. Mater..

[29] İlbay Z., Şahin S., Kerkez Ö., Bayazit S. (2015). Isolation of naproxen from wastewater using carbon-based magnetic adsorbents. Int. J. Environ. Sci. Technol..

[30] Wong K.T., Yoon Y., Snyder S.A., Jang M. (2016). Phenyl-functionalized magnetic palm-based powdered activated carbon for the effective removal of selected pharmaceutical and endocrine-disruptive compounds. Chemosphere.

[31] Arya V., Philip L. (2016). Adsorption of pharmaceuticals in water using Fe3O4 coated polymer clay composite. Microporous Mesoporous Mater..

[32] Danalıoğlu S.T., Bayazit Ş.S., Kerkez Kuyumcu Ö., Salam M.A. (2017). Efficient removal of antibiotics by a novel magnetic adsorbent: Magnetic activated carbon/chitosan (MACC) nanocomposite. J. Mol. Liq..

[33] Castro C.S., Guerreiro M.C., Gonçalves M., Oliveira L.C.A., Anastácio A.S. (2009). Activated carbon/iron oxide composites for the removal of atrazine from aqueous medium. J. Hazard. Mater..

[34] Brunauer S., Emmett P.H., Teller E. (1938). Adsorption of Gases in Multimolecular Layers. J. Am. Chem. Soc..

[35] Marsh H., Rand B. (1970). The Characterization of Microporous Carbons by Means of the Dubinin-Radushkevich Equation. J. Colloid Interface Sci..

[36] Calisto V., Domingues M.R.M., Erny G.L., Esteves V.I. (2011). Direct photodegradation of carbamazepine followed by micellar electrokinetic chromatography and mass spectrometry. Water Res..

[37] Hassan M., Naidu R., Du J., Liu Y., Qi F. (2020). Critical review of magnetic biosorbents: Their preparation, application, and regeneration for wastewater treatment. Sci. Total Environ..

[38] Lago R.M., Sapag K., Fabris J.D., Rios R.V.R.A., Oliveira L.C.A., Garg V. (2003). Activated carbon/iron oxide magnetic composites for the adsorption of contaminants in water. Carbon.

[39] Reguyal F., Sarmah A.K., Gao W. (2017). Synthesis of magnetic biochar from pine sawdust via oxidative hydrolysis of FeCl2 for the removal sulfamethoxazole from aqueous solution. J. Hazard. Mater..

[40] Wang S.Y., Tang Y.K., Li K., Mo Y.Y., Li H.F., Gu Z.Q. (2014). Combined performance of biochar sorption and magnetic separation processes for treatment of chromium-contained electroplating wastewater. Bioresour. Technol..

[41] Wong K.T., Yoon Y., Jang M. (2015). Enhanced recyclable magnetized palm shell waste-based powdered activated carbon for the removal of ibuprofen: Insights for kinetics and mechanisms. PLoS ONE.

[42] Wan J., Deng H.P., Shi J., Zhou L., Su T. (2014). Synthesized magnetic manganese ferrite nanoparticles on activated carbon for sulfamethoxazole removal. Clean-Soil Air Water.

[43] Cornell R.M., Schwertmann U. (2003). The Iron Oxides Structure, Properties, Reactions, Occurences and Uses.

[44] Thines K.R., Abdullah E.C., Mubarak N.M., Ruthiraan M. (2017). Synthesis of magnetic biochar from agricultural waste biomass to enhancing route for waste water and polymer application: A review. Renew. Sustain. Energy Rev..

[45] Ma X., Yang H., Yu L., Chen Y., Li Y. (2014). Preparation, Surface and Pore Structure of High Surface Area Activated Carbon Fibers from Bamboo by Steam Activation. Materials.

[46] Velo-gala I., López-Peñalver J.J., Sánchez-Polo M., Rivera-Utrilla J. (2014). Surface modifications of activated carbon by gamma irradiation. Carbon.

[47] Rattanachueskul N., Saning A., Kaowphong S., Chumha N., Chuenchom L. (2017). Magnetic carbon composites with a hierarchical structure for adsorption of tetracycline, prepared from sugarcane bagasse via hydrothermal carbonization coupled with simple heat treatment process. Bioresour. Technol..

[48] Yamashita T., Hayes P. (2008). Analysis of XPS spectra of Fe2+ and Fe3+ ions in oxide materials. Appl. Surf. Sci..

[49] Kakavandi B., Esrafili A., Mohseni-Bandpi A., Jafari A.J., Kalantary R.R. (2014). Magnetic Fe_3_O_4_@C nanoparticles as adsorbents for removal of amoxicillin from aqueous solution. Water Sci. Technol..

[50] Singh K.P., Singh A.K., Singh U.V., Verma P. (2012). Optimizing removal of ibuprofen from water by magnetic nanocomposite using Box-Behnken design. Environ. Sci. Pollut. Res..

[51] Shan D., Deng S., Zhao T., Wang B., Wang Y., Huang J., Yu G., Winglee J., Wiesner M.R. (2016). Preparation of ultrafine magnetic biochar and activated carbon for pharmaceutical adsorption and subsequent degradation by ball milling. J. Hazard. Mater..

