# Supplementary material for: Producing Magnetic Nanocomposites from Paper Sludge for the Adsorptive Removal of Pharmaceuticals from Water—A Fractional Factorial Design
Source: Nanomaterials (Basel). 2021 Jan 22;11(2):287. doi: 10.3390/nano11020287 (PMC7911794; doi:10.3390/nano11020287)
Supplement: Supplementary file 1 [file nanomaterials-11-00287-s001.pdf]

SUPPLEMENTARY MATERIAL

Producing magnetic nanocomposites from paper sludge for the adsorptive removal of pharmaceuticals from water - A fractional factorial design

Luciana S. Rocha[1,*], Érika M.L. Sousa[1], María V. Gil[2], João A.B.P. Oliveira[1], Marta Otero[3], Valdemar I. Esteves[1], Vânia Calisto[1]

[1] *Department of Chemistry and CESAM, University of Aveiro, 3810-193 Aveiro, Portugal*

[2] *Instituto de Ciencia y Tecnología del Carbono, INCAR-CSIC, Francisco Pintado Fe 26, 33011 Oviedo, Spain*

[3] *Department of Environment and Planning and CESAM, University of Aveiro, 3810-193 Aveiro, Portugal*

Table S1. Physical and chemical properties of amoxicillin tri-hydrate (AMX), carbamazepine (CBZ) and sodium diclofenac (DCF).

Table S2. Values obtained for the specific surface area (S_{BET}) and textural properties (total pore volume V_p, micropore volume V_{mic}, and average pore diameter D) of PAC and the eighteen MACs.

Table S3. Results of ANOVA analysis: sum of square (SS) and mean of square (MS) values, degree of freedom (df), F-test and the p-value (confidence level of 95%).

Table S4. Values of A (%) obtained for AMX, CBZ and DCF using MACs prepared using different carbon precursors, along with the experimental conditions used in the adsorption experiments (C_i of each pharmaceutical, dose of MAC, pH, temperature and contact time).

Figure S1. N_2 adsorption isotherms expressed as (A.) adsorption (cm^3 STP g^{-1}) *vs* relative pressure (p/p^0) and (B.) adsorption (cm^3 STP g^{-1}) *vs* -log (p/p^0) of the following materials: PAC (▬), MAC 4 (▬), MAC 7 (▬), MAC 11 (▬) and MAC 17 (▬).

Figure S2. Pore size distribution of PAC, MAC 3, MAC6, MAC 13 and MAC 16.

Figure S3. Values obtained for S_{BET} (A.) and M_s (B.) as a function of PAC:Fe salts ratio (*w*/*w*).

Figure S4. SEM images of the MAC 7 using a magnification of 20000 (A.) and 40000× (B.).

*Corresponding author: lrocha@ua.pt

Figure S5. Overall XPS spectra of PAC (▬), MAC 4 (▬), MAC 7 (▬), MAC 11 (▬) and MAC 17 (▬).

Figure S6. Deconvolution of C1s, O1s and Fe2p XPS peaks of MAC 7, MAC 11 and MAC 17: experimental peak (—), adjusted peak (—) and the component groups (—).

*Corresponding author: lrocha@ua.pt

Table S1. Physical and chemical properties of amoxicillin tri-hydrate (AMX) [1], carbamazepine (CBZ) [2] and sodium diclofenac (DCF) [3].

Pharmaceutical		
Classe	Molecular structure	Properties
Antibiotic	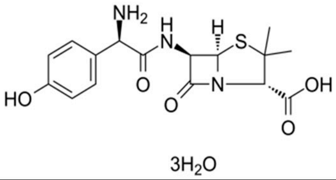	**Amoxicillin tri-hydrate:**<br><br>Mw: 419.5 g mol^{-1}<br>pk_{a1}: 3.23, pk_{a2}: 7.43<br>log K_{ow}: 0.87
Antiepileptic	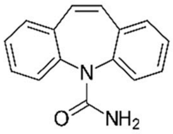	**Carbamazepine:**<br><br>Mw: 236.3 g mol^{-1}<br>pk_a:13.9<br>log K_{ow}: 2.45
Non-steroidal anti-inflammatory drug	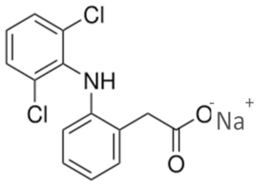	**Sodium diclofenac:**<br><br>Mw: 318.1 g mol^{-1}<br>pk_a: 4.15<br>log K_{ow}: 4.51

[1] https://pubchem.ncbi.nlm.nih.gov/compound/Amoxicillin-trihydrate (accessed in 15.07.2020)

[2] https://pubchem.ncbi.nlm.nih.gov/compound/Carbamazepine (accessed in 15.07.2020)

[3] https://pubchem.ncbi.nlm.nih.gov/compound/Diclofenac-sodium (accessed in 15.07.2020)

Table S2. Values obtained for the specific surface area (S_{BET}) and textural properties (total pore volume V_p, micropore volume V_{mic}, and average pore diameter D) of PAC and the eighteen MACs.

Material	N_2 adsorption at −196 °C			
	S_{BET} (m^2 g^{-1})	V_p (cm^3 g^{-1})	V_{mic} (cm^3 g^{-1})	D (nm)
PAC	1438	1.00	0.57	1.39
MAC1	782	0.68	0.31	1.74
MAC2	658	0.56	0.26	1.69
MAC3	532	0.46	0.21	1.73
MAC4	725	0.63	0.29	1.73
MAC5	653	0.56	0.26	1.70
MAC6	475	0.48	0.19	2.03
MAC7	794	0.74	0.32	1.87
MAC8	609	0.60	0.24	1.97
MAC9	672	0.62	0.27	1.86
MAC10	624	0.58	0.25	1.87
MAC11	741	0.76	0.30	2.04
MAC12	552	0.59	0.22	2.15
MAC13	828	0.86	0.33	2.08
MAC14	645	0.68	0.26	2.10
MAC15	538	0.57	0.21	2.13
MAC16	899	0.82	0.36	1.83
MAC17	767	0.72	0.31	1.87
MAC18	641	0.68	0.26	2.13

Table S3. Results of ANOVA analysis: sum of square (SS) and mean of square (MS) values, degree of freedom (df), F-test and the *p*-value (confidence level of 95%).

	Source	*SS*	df	*MS*	*F*	*p*-values*
S_{BET} ($m^2\ g^{-1}$)	χ_1	2.47×10^4	2	1.24×10^4	2.29	0.152
	χ_2	1.43×10^5	2	7.17×10^4	13.3	**0.002**
	χ_3	4.23×10^3	2	2.11×10^3	0.39	0.686
	χ_4	3.89×10^3	1	3.89×10^3	0.72	0.416
M_s (emu g^{-1})	χ_1	2.09×10^1	2	1.04×10^1	0.17	0.846
	χ_2	1.26×10^3	2	6.32×10^2	10.3	**0.004**
	χ_3	2.02×10^2	2	1.01×10^2	1.64	0.241
	χ_4	7.31×10^1	1	7.31×10^1	1.19	0.301
A_{AMX} (%)	χ_1	3.30×10^2	2	1.65×10^2	1.79	0.217
	χ_2	3.74×10^2	2	1.87×10^2	2.03	0.182
	χ_3	1.11×10^1	2	5.56	0.06	0.942
	χ_4	6.50×10^1	1	6.50×10^1	0.70	0.421
A_{CBZ} (%)	χ_1	2.39×10^2	2	1.19×10^2	1.2	0.342
	χ_2	1.92×10^3	2	9.58×10^2	9.59	**0.005**
	χ_3	1.09×10^1	2	5.44	0.05	0.947
	χ_4	1.20×10^2	1	1.20×10^2	1.2	0.299
A_{DCF} (%)	χ_1	2.36×10^1	2	1.18×10^1	0.29	0.757
	χ_2	7.86×10^2	2	3.93×10^2	9.51	**0.005**
	χ_3	1.94×10^2	2	9.72×10^1	2.35	0.146
	χ_4	3.74	1	3.47	0.08	0.778

* The bold *p*-values indicate the significant effect of factor on the response.

Table S4. Values of A (%) obtained for AMX, CBZ and DCF using MACs prepared using different carbon precursors, along with the experimental conditions used in the adsorption experiments (C_i of each pharmaceutical, dose of MAC, pH, temperature and contact time).

Carbon Precursor	Adsorption experiments	A (%)	Reference
	AMX		
Commercial PAC	C_{AMX}=50 mg L^{-1}; Dose $_{MAC}$=1000 mg L^{-1}<br>pH=5; T=20 ºC<br>Contact time: 1.5 h	95	[49]
Waste-based PAC	C_{AMX}=5 mg L^{-1}; Dose $_{MAC}$=35 mg L^{-1};<br>pH=6 (a); T=25 ºC<br>Contact time: 4 h	61-70	Present study<br>(MAC 4, 7, 11, 17)
	CBZ		
Commercial PAC	C_{CBZ}=5.88 mg L^{-1}; Dos $_{MAC}$= 248.5 mg L^{-1};<br>pH=6.6 (b); T=25 ºC<br>Contact time: 0.136 h	93	[25]
Commercial granular AC	C_{CBZ}=30 mg L^{-1}; Dose $_{MAC}$=200 mg L^{-1}<br>pH=6<br>Contact time: 48 h	~80	[36]
Waste-based PAC	C_{CBZ}=5 mg L^{-1}; Dose $_{MAC}$=35 mg L^{-1}<br>pH=6 (a); T=25 ºC<br>Contact time: 4 h	69-77	Present study<br>(MAC 4, 7, 11, 17)
	DCF		
Waste-based PAC	C_{DCF}=5 mg L^{-1}; Dose $_{MAC}$=35 mg L^{-1}<br>pH=6 (a); T=25 ºC<br>Contact time: 4 h	80-84	Present study<br>(MAC 4, 7, 11, 17)

(a) Unadjusted value. (b) The adsorption experiments were performed in wastewater treatment plant influent.

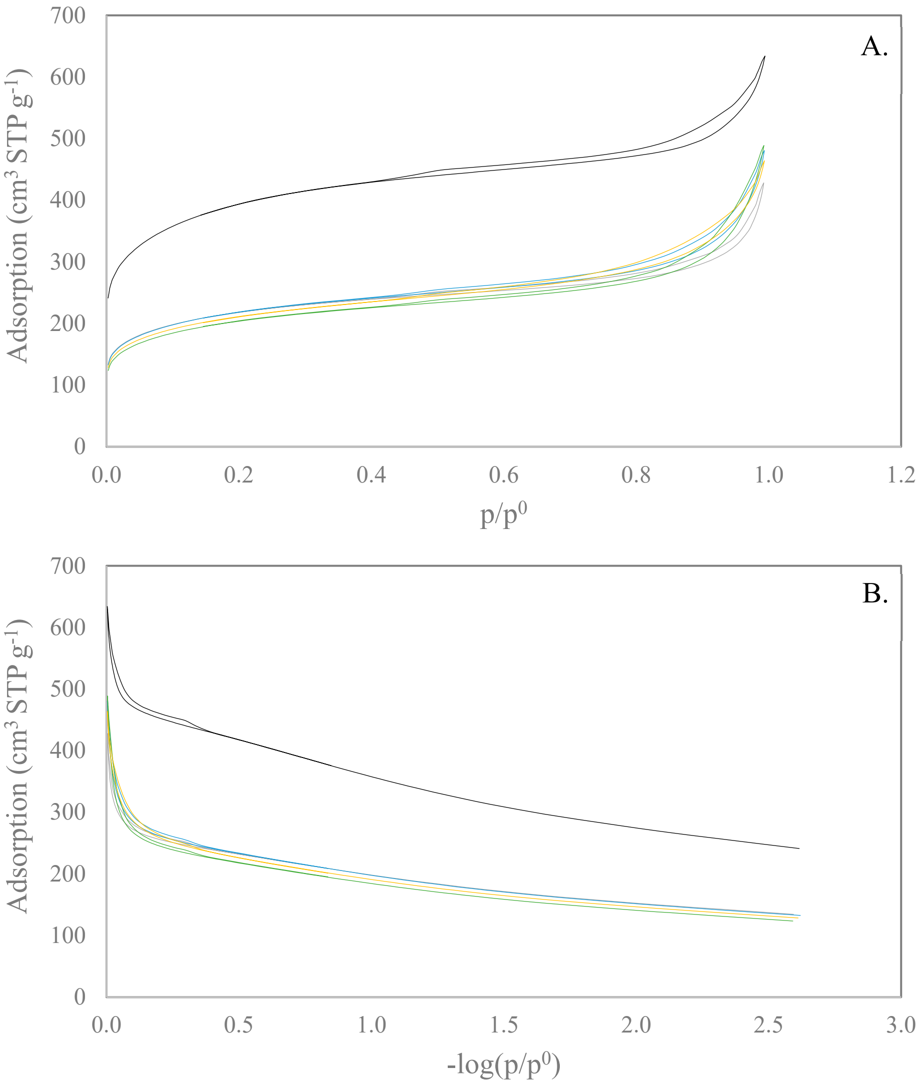

Figure S1. N_2 adsorption isotherms expressed as (A) adsorption (cm^3 STP g^{-1}) *vs* relative pressure (p/p^0) and (B) adsorption (cm^3 STP g^{-1}) *vs* -log (p/p^0) of the following materials: PAC (▬), MAC 4 (▬), MAC 7 (▬), MAC 11 (▬) and MAC 17 (▬).

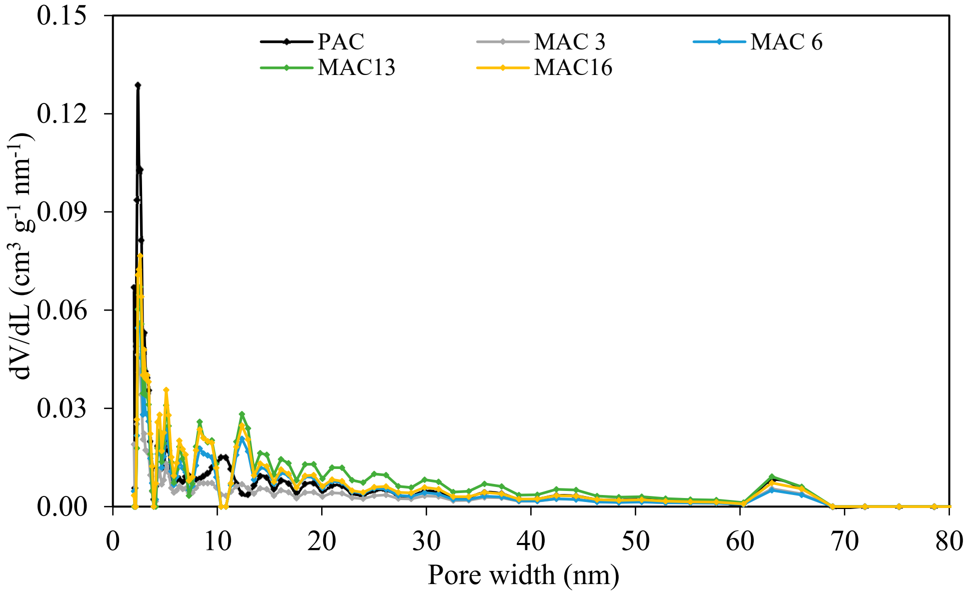

Figure S2. Pore size distribution of PAC, MAC 3, MAC6, MAC 13 and MAC 16.

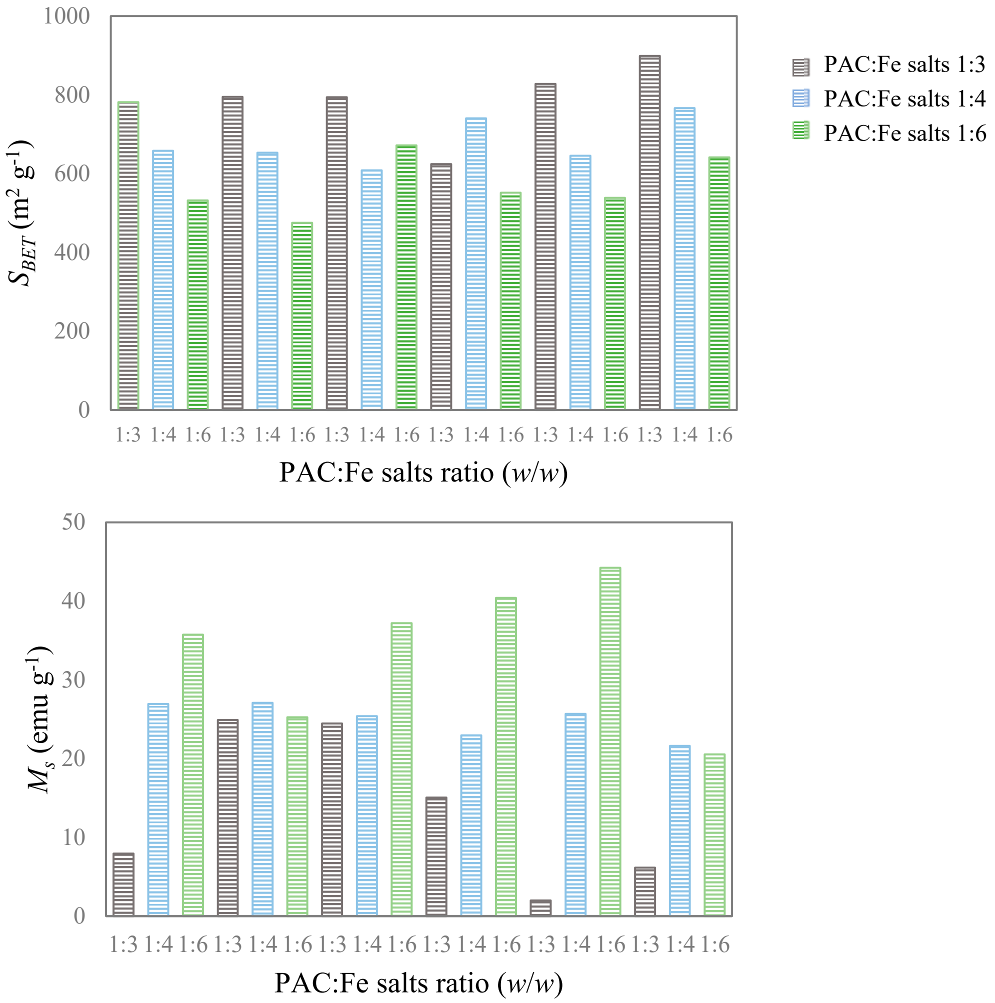

Figure S3. Values obtained for S_{BET} (A.) and M_s (B.) as a function of PAC:Fe salts ratio (*w*/*w*).

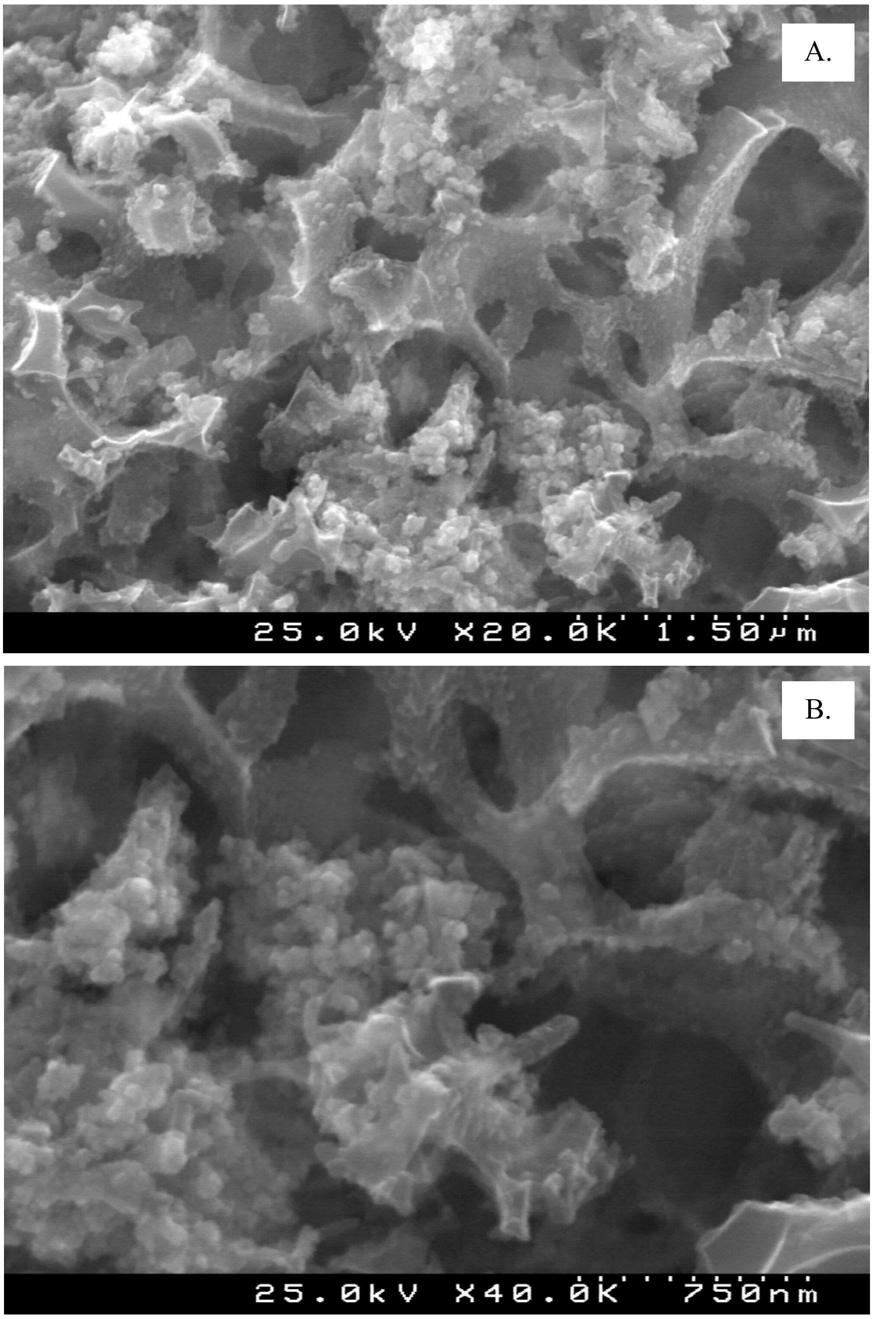

Figure S4. SEM images of the MAC 7 using a magnification of 20000 (A.) and 40000× (B.).

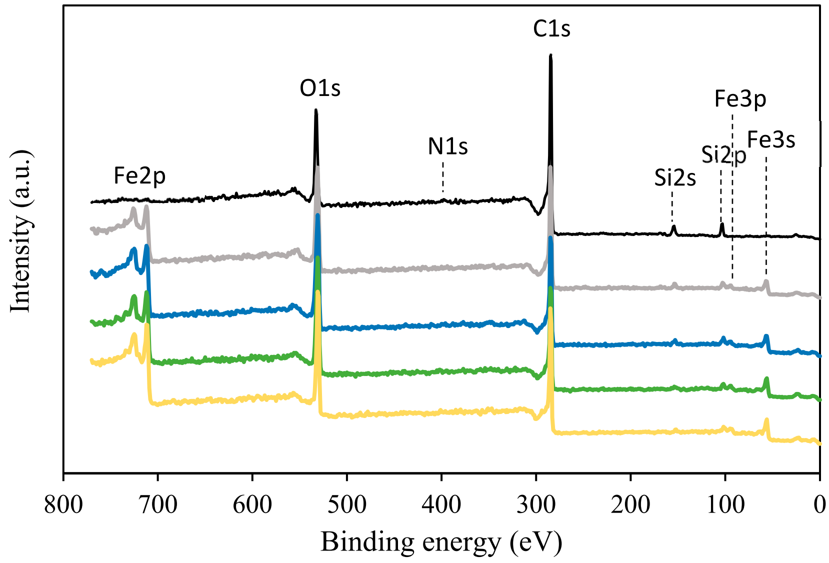

Figure S5. Overall XPS spectra of PAC (▬), MAC 4 (▬), MAC 7 (▬), MAC 11 (▬) and MAC 17 (▬).

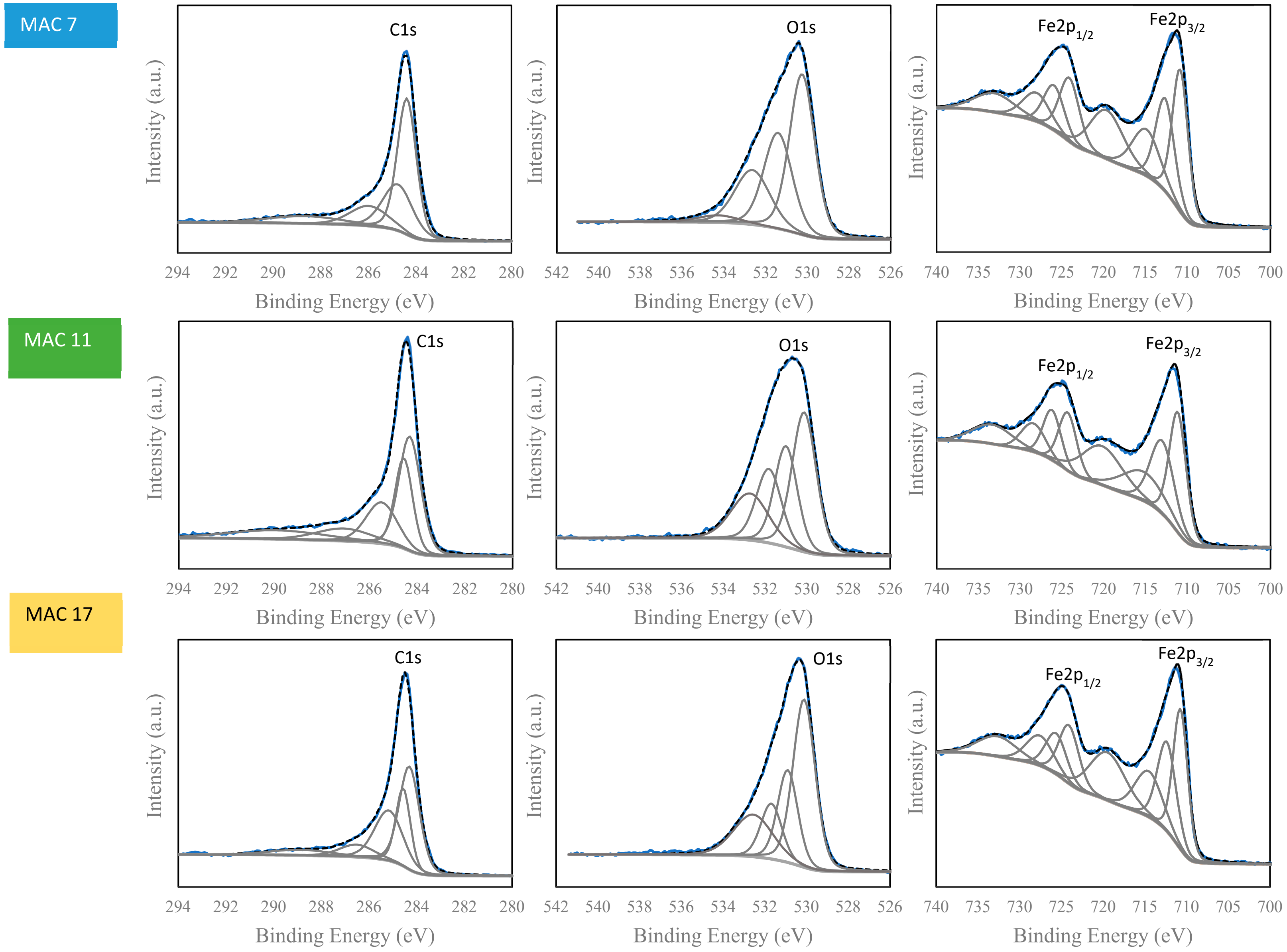

Figure S6. Deconvolution of C1s, O1s and Fe2p XPS peaks of MAC 7, MAC 11 and MAC 17: experimental peak (—), adjusted peak (—) and the component groups (—).
